# Novel Axl-driven signaling pathway and molecular signature characterize high-grade ovarian cancer patients with poor clinical outcome

**DOI:** 10.18632/oncotarget.5087

**Published:** 2015-09-04

**Authors:** Katia Rea, Patrizia Pinciroli, Marialuisa Sensi, Federica Alciato, Brigitte Bisaro, Ludmila Lozneanu, Francesco Raspagliesi, Floriana Centritto, Sara Cabodi, Paola Defilippi, Gian Carlo Avanzi, Silvana Canevari, Antonella Tomassetti

**Affiliations:** ^1^ Unit of Molecular Therapies, Department of Experimental Oncology and Molecular Medicine, Fondazione IRCCS Istituto Nazionale dei Tumori, Milan, Italy; ^2^ Functional Genomics and Bioinformatics Core Facility, Department of Experimental Oncology and Molecular Medicine, Fondazione IRCCS Istituto Nazionale dei Tumori, Milan, Italy; ^3^ Department of Traslational Medicine, Università degli Studi del Piemonte Orientale, Novara, Italy; ^4^ Department of Molecular Biotechnology and Health Sciences, University of Torino, Italy; ^5^ Department of Morphofunctional Sciences, Histology, Morphopatology, “Grigore T. Popa” University of Medicine and Pharmacy, Iassy, Romania; ^6^ Gynecology Oncology Unit, Department of Surgery, Fondazione IRCCS Istituto Nazionale dei Tumori, Milan, Italy

**Keywords:** Axl, Gas6, ovarian cancer, p130Cas, extracellular matrix

## Abstract

High-grade epithelial ovarian cancer (HGEOC) is a clinically diverse and molecularly heterogeneous disease comprising subtypes with distinct biological features and outcomes. The receptor tyrosine kinases, expressed by EOC cells, and their ligands, present in the microenvironment, activate signaling pathways, which promote EOC cells dissemination. Herein, we established a molecular link between the presence of Gas6 ligand in the ascites of HGEOCs, the expression and activation of its receptor Axl in ovarian cancer cell lines and biopsies, and the progression of these tumors. We demonstrated that Gas6/Axl signalling converges on the integrin β3 pathway in the presence of the adaptor protein p130Cas, thus inducing tumor cell adhesion to the extracellular matrix and invasion. Accordingly, Axl and p130Cas were significantly co-expressed in HGEOC samples. Clinically, we identified an Axl-associated signature of 62 genes able to portray the HGEOCs with the shortest overall survival. These data biologically characterize a group of HGEOCs and could help guide a more effective therapeutic approach to be taken for these patients.

## INTRODUCTION

Epithelial ovarian cancer (EOC) is the fifth leading cause of cancer-related deaths among women and of death among all gynecologic tumors [1]. In spite of progress in its diagnosis and treatment, EOC incidence and mortality rates have been mostly unchanged over the last 30 years [2]. The most recent EOC classification identifies genetically stable, low-grade type I tumors, and high-grade (HGEOC) type II tumors, characterized by a high chromosomal instability and intra-tumor heterogeneity [3]. HGEOC, fallopian tubal, and peritoneal carcinomas are considered a single clinical entity due to their shared clinical behavior and treatment; however, HGEOC is a morphological and molecularly heterogeneous disease, and a rational approach for optimal therapeutic regimens has not yet been provided. In the most recent years, gene expression profiles have been widely exploited to better characterize the biology of these tumors and to generate a patient classification aimed at identifying the most efficacious therapeutic approach to take [4–8].

HGEOCs rarely metastasize to distant sites, but instead disseminate over the surface of the peritoneum [9]. The dissemination process occurs when a single tumor cell or small clusters are shed from the primary site or from peritoneal secondary lesions. These floating multicellular cancer aggregates (MCAs) overcome anoikis, persist in ascites, and then re-adhere on the surface of the peritoneum or omentum, suggesting that the attachment of cancer cells to the basement membrane is a key step in HGEOC dissemination.

These processes require the contribution of cell adhesion molecules and receptor tyrosine kinases (RTKs), expressed by EOC cells, and their ligands, present in the tumor microenviroment. Among RTKs, Axl, belonging to the TAM RTK family together with Mer and Tyro3, is frequently overexpressed in several human solid tumors, and can influence tumorigenicity, invasiveness, metastasis formation, or resistance to therapeutic agents [10]. Treatment with a soluble Axl moiety, which is able to inhibit the binding of one of its ligands, Gas6, to the membrane-bound Axl, has been shown to reduce the peritoneal metastatic EOC burden in mice [11]. Accordingly, Axl knockdown in ovarian cancer SKOV3 cells has been shown to reduce PI3K/Akt activation and the expression of MMP-1 and MMP-9, both *in vitro* and *in vivo* using the peritoneal xenograft model of ovarian cancer [11].

The natural ligand of Axl is the 75 kDa vitamin K-dependent protein Gas6 [12]. Gas6 is physiologically involved in a wide range of cellular responses, including cell survival, angiogenesis, phagocytosis, platelet aggregation, vascular biology, inflammation, and immunity [12]. Soluble Gas6 is also present in plasma at a concentration of around 20–50 ng/mL (0.25 nmol/L) [13]. In solid tumors, Gas6 promotes the proliferation of prostate cancer cells [14].

The oncogenic nature of Axl is demonstrated through its activation of the signaling pathways involved in the proliferation, migration, and inhibition of apoptosis, and in therapeutic resistance [15]. In melanomas, Axl gene expression is associated with the most invasive tumors and with resistance to BRAF inhibitors [16, 17]. The presence of Gas6 in the human HGEOC microenvironment, the molecular processes activated downstream of Gas6-stimulated HGEOC cells, and the impact of this signaling cascade on HGEOC patients' outcomes have not been so far assessed. Therefore, herein, we aimed to investigate the signaling cascade activated by the Gas6/Axl axis and to evaluate the clinical relevance of Axl expression.

## RESULTS

### Gas6 is expressed in EOC cells and activates the TAM RTK Axl

**Figure 1:**
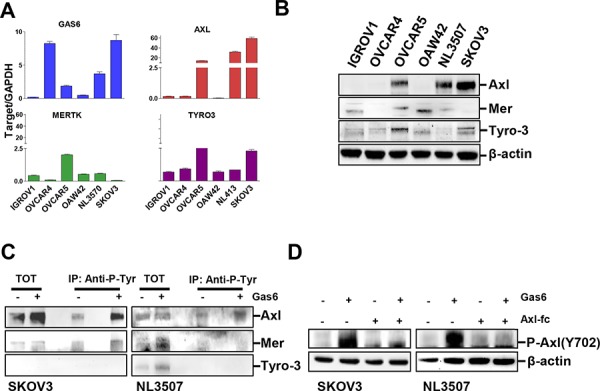
Gas6 is expressed in EOC cells and activates the TAM RTK Axl **A.** Real-time PCR showing the levels of mRNA for Gas6, AXL, MERTK, and TYRO-3 in six EOC cell lines. Results are presented as relative expression normalized to GAPDH mRNA levels. **B.** Western blotting on the total cell lysates from the same six EOC cell lines. **C.** IP with Anti-P-Tyrosine (P-Tyr) performed on lysates from starved or Gas6-stimulated SKOV3 and NL3507 cells. Immunoprecipitated samples were analyzed by western blotting with Abs against the proteins reported on the right. **D.** Western blotting on the total cell lysates from starved SKOV3 and NL3507 cells pre-treated with Axl-Fc (2.5 μg/ml) and stimulated or not with Gas6 (500 ng/ml). Abs are reported on the right. β-actin was used as the gel loading control.

We first checked by real time RT-PCR the expression of Gas6 and Axl, Mer, and Tyro-3 in a panel of human ovarian cancer cell lines. OVCAR4 and SKOV3 cells expressed the highest levels of Gas6 (Fig. 1A), while OVCAR5 and NL3507 barely showed detectable Gas6 transcript. The expression of TAM receptors was heterogeneous, Axl being expressed at high levels in OVCAR5, NL3507, and SKOV3 cells, in both the RT-PCR and western blotting experiments (Fig. 1A and 1B). Mer and Tyro-3 were expressed at low levels in all cell lines (Fig. 1A and 1B); slightly higher levels of Mer were observed in IGROV1, OVCAR5, OAW42, and NL3507, while Tyro3 expression was found to be higher in OVCAR5 and SKOV3.

To assess which TAM receptors were activated by Gas6 stimulation, immunoprecipitation (IP) with an anti-phosphotyrosine (p-Tyr) antibody (Ab) was performed on starved SKOV3 cells (expressing Axl and Tyro-3), and on starved NL3507 cells (expressing Axl and Mer), with and without stimulation with Gas6 (500 ng/ml). In both cell lines, Axl was basally slightly phosphorylated, probably by endogenously produced Gas6 (see Fig. 1A), while Gas6 stimulation induced increased levels of Axl phosphorylation (Fig. 1C). In SKOV3 cells, a slight amount of phosphorylated Tyro-3 was observed, while in NL3507 Mer and Tyro-3 were not phosphorylated upon Gas6 stimulation (Fig. 1C). None of the TAM receptors was immunoprecipitated with a mouse anti-serum (Supplementary Fig. 1). In both Gas6-stimulated cell lines, phosphorylation of Axl was inhibited by the presence of the recombinant Axl-Fc protein (Fig. 1D).

These data demonstrate that the Gas6 stimulation of ovarian cancer cells activates RTK Axl.

### Gas6-stimulated promotion of invasion through the interaction between ovarian cancer cells and ECM

**Figure 2:**
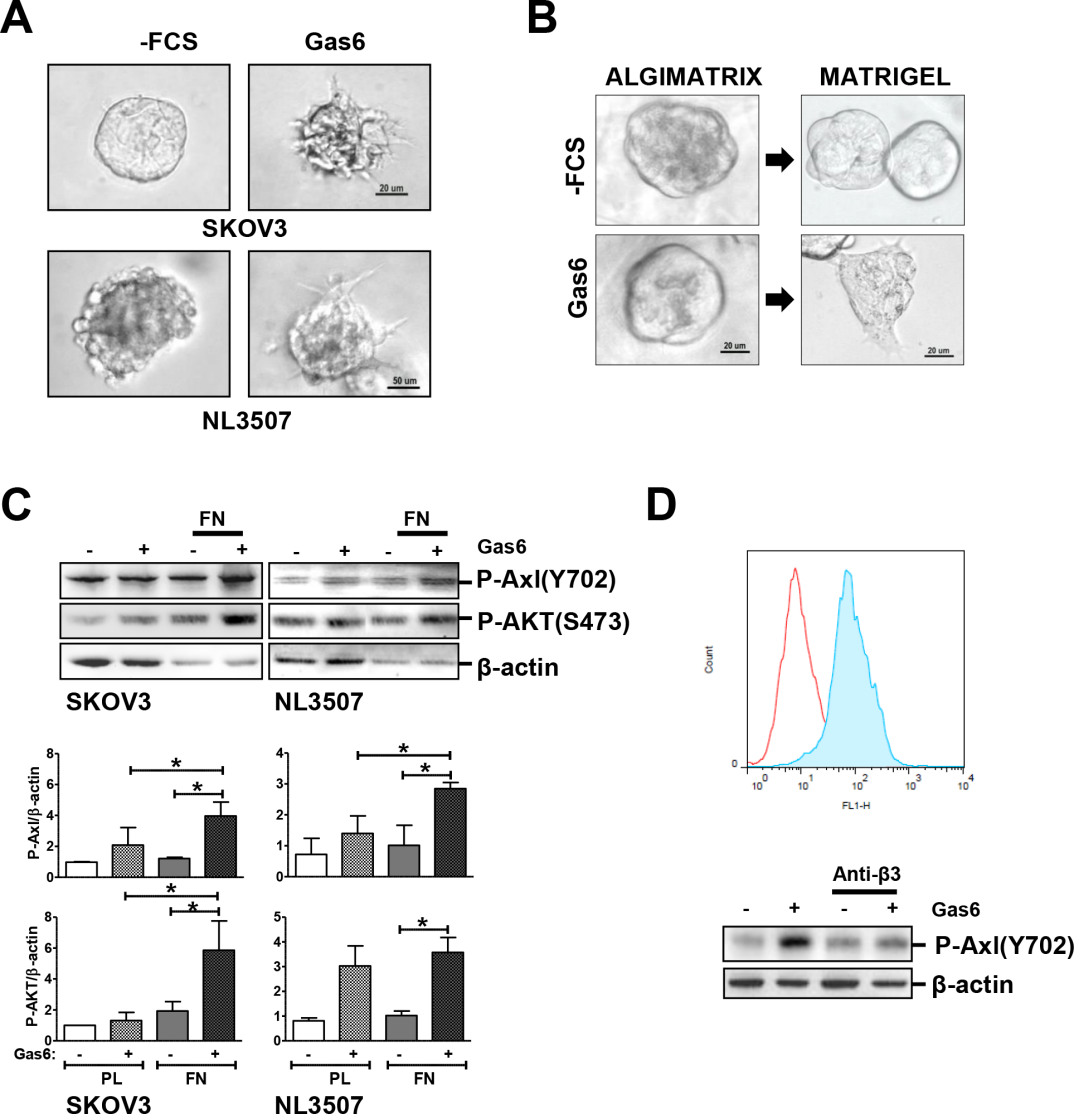
Gas6-stimulated promotion of invasion through the interaction between ovarian cancer cells and ECM **A.** Invasion assay of starved or Gas6-stimulated SKOV3 and NL3507 cells grown in Matrigel. Representative images of three independent experiments are shown. **B.** Left panel, phase contrast microscopy of starved or Gas6-stimulated SKOV3 cells grown and stimulated with Gas6. **C.** Upper panel, western blotting on the total cell lysates from starved or Gas6-stimulated SKOV3 and NL3507 cells seeded on FN for 30 min. A representative experiment of the three performed is shown. Abs are reported on the right. β-actin was used as the gel loading control. Lower panel, quantitative evaluation of phosphorylated Axl in Gas6-stimulated cells upon adhesion on FN. The graph reports the mean ± SD from the three independent experiments. **D.** Upper panel, membrane staining of the integrin β3 receptor determined by flow cytometry on the NL3507 cell line. The expression of the other integrin β receptors on both the SKOV3 and NL3505 cells was also evaluated and the results are reported in Supplementary Fig. 3. The red and light blue peaks, respectively, represent the fluorescence of the cells incubated with the secondary antibody alone as control (α-mouse) and the anti-integrin β3 Ab (Anti-β3). Lower panel, western blotting on the total cell lysates from starved or Gas6-stimulated NL3507 cells, in the absence or in the presence of the anti-integrin β3 Ab. A representative experiment is shown. Abs are reported on the right. β-actin was used as the gel loading control.

Next, we assessed whether Gas6 stimulation induced ovarian cancer cell invasion. We cultured SKOV3 and NL3507 cells in reduced growth factor Matrigel-embedded 3D to drive the formation of spheroids, in order to mimic the invasion process occurring *in vivo*. After 5 days, single cells from both cell lines were able to form spheroids in Matrigel (Fig. 2A, left panels). The spheroids were serum-starved and were tested stimulated and unstimulated with Gas6 to evaluate its effect on cell behavior. Gas6 stimulation induced the formation of a stellate invasive phenotype characterized by invadopodia and chains of cells invading the surrounding Matrigel, reminiscent of a collective cell invasion (Fig. 2A, right panel). To test whether the interaction with a component of the ECM was essential for Gas6-induced invasion, we used an inert 3D alginate scaffold, the AlgiMatrix™, with which human integrins do not interact [18]. SKOV3 spheroids, grown in AlgiMatrix™ for 12 days, were serum starved and stimulated or not with Gas6. In contrast to what we observed before, in the absence of Matrigel, Gas6 did not induce any SKOV3 spheroids morphological changes (Fig. 2B, left panel). AlgiMatrix™-cultured SKOV3 spheroids were then embedded in Matrigel (as described in the scheme displayed in Supplementary Fig. 2), and stimulated or not with Gas6, as above. As observed in panel A, an invasive morphology was observed only in the presence of Gas6 (Fig. 2B, right panel).

**Figure 3:**
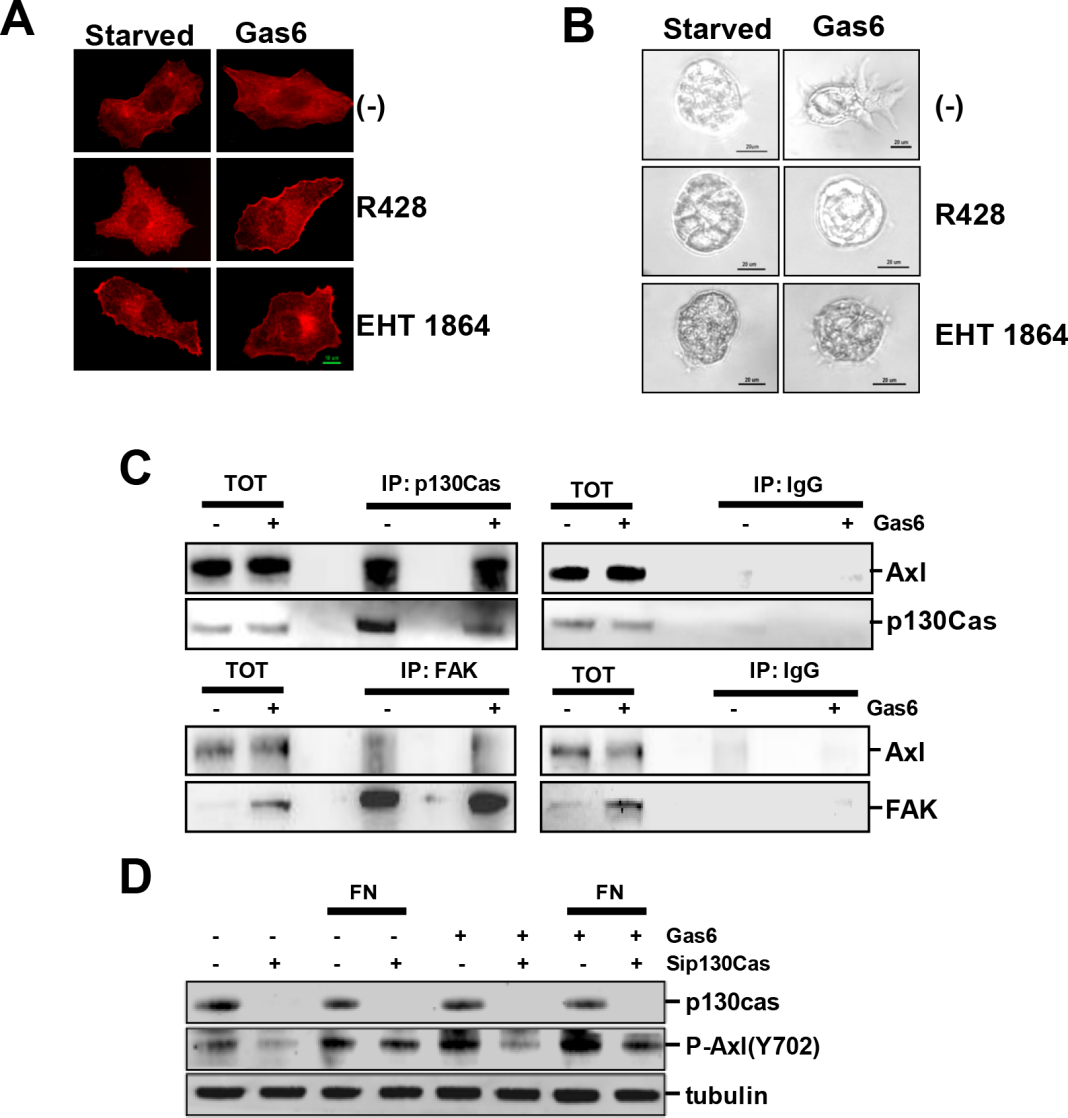
Gas6/Axl signaling triggers PI3K/AKT/rac activation and requires the scaffold protein p130Cas **A.** IF performed on SKOV3 cells grown on FN, starved and then induced to migrate through a wound in the presence or not of Gas6. Cells were treated with the reported inhibitors, and F-actin was stained with phalloidin (red). **B.** Invasion assay of starved or Gas6-stimulated SKOV3 and NL3507 cells grown in Matrigel. Cells were treated with the reported inhibitors. Representative images of the three independent experiments are shown. **C.** IP performed with anti-p130Cas (upper panel) or with anti-FAK (lower panel), respectively, on lysates of starved or Gas6-stimulated SKOV3 cells. Normal mouse or rabbit (IgG) sera were used as the negative control, respectively. The immunoprecipitated samples were analyzed by western blotting. **D.** Western blotting on the total cell lysates from SKOV3 cells transiently transfected with a control siRNA (−) or with a pool of siRNA against p130Cas (sip130Cas). SiRNA-transfected SKOV3 cells were starved or Gas6-stimulated and seeded on FN for 30 min. Immunoblottings were performed with Abs against the proteins reported on the right. Alpha (tubulin) was used as the gel loading control.

These results indicate that Gas6 stimulation alone is not sufficient to induce changes in SKOV3 cell morphology but the interaction of Gas6-stimulated cells with ECM induces cellular changes. To assess the contribution of ECM proteins to Axl activation by Gas6, starved SKOV3 and NL3507 cells were allowed to adhere for 30 min on plastic or fibronectin (FN)-coated dishes, in the presence or absence of Gas6. Axl activation was assessed in total cell lysates by western blotting. The Gas6 stimulation of FN adherent cells induced about two fold Axl phosphorylation levels in respect to the plastic adherent cells (Fig. 2C). Since Axl phosphorylation should trigger PI3K/AKT activation [11], we also observed a higher AKT phosphorylation upon Gas6 stimulation. Both FN adherent SKOV3 and NL3507 cells showed AKT phosphorylation significantly higher with respect to the cells grown on plastic (Fig. 2C, lower panel). Among the different integrin β chain molecules, SKOV3 and NL3507 cells express β3, as assessed by FACS analysis (Fig. 2D, upper panel, and Supplementay Fig. 3). To assess whether β3 integrin was involved in Gas6-stimulated Axl activation, NL3507 cells were allowed to adhere on FN-coated dishes for 30 min in the presence of Ab able to inhibit integrin β3/FN interaction. As expected, by preventing the binding of integrin β3 to the ECM, Gas6-induced Axl tyrosine phosphorylation was completely eliminated (Fig. 2D, lower panel).

These data clearly show, for the first time, that the Gas6-stimulated Axl signaling pathway is dependent on the adhesion of ovarian cancer cells to the ECM through the integrin β3.

### Gas6/Axl signaling triggers PI3K/AKT/rac activation and requires the scaffold protein p130Cas

In order to assess the biochemical and cellular mechanisms activated in ovarian cancer cells by Gas6, SKOV3 cells were Gas6 stimulated while adhering to FN, and the actin assembly was evaluated by immunofluorescence (IF) with labeled phalloidin. Gas6-induced protrusive structures at the leading edge were associated with the presence of stress fibers (Fig. 3A). In the presence of R428, an inhibitor of Axl phosphorylation, actin nucleation was inhibited, as well as the formation of stress fibers. Interestingly, the treatment of FN-adherent SKOV3 cells with the rac inhibitor EHT1864 recapitulated the effect of the Axl inhibitor on actin assembly and the formation of stress fibers.

These results were further supported by inhibition of the invasion of Gas6-stimulated SKOV3 spheroids in Matrigel by both the Axl and rac inhibitors (Fig. 3B). Furthermore, the treatment of SKOV3 cells with the src inhibitor PP2 did not inhibit Gas6-induced Matrigel invasion (data not shown).

The synergism between RTKs and integrins is mediated by intracellular scaffold proteins, such as FAK and p130Cas, which integrate both signalings leading to the intracellular response [19]. To study if these scaffold proteins could be the mediator(s) between Gas6/Axl and ECM, the possible interaction between Axl and p130Cas or FAK was analyzed by IP, as performed on starved SKOV3 stimulated or not with Gas6. Total cell lysates were immunoprecipitated with anti-p130Cas (Fig. 3C, upper panel) or-FAK Abs (lower panel) and the obtained protein complexes were analyzed by western blotting with anti-Axl Ab. Axl co-immunoprecipitated with the p130Cas scaffold protein in the absence and in the presence of Gas6 (Fig. 3C, upper panel), while it only slightly interacted with FAK (lower panel).

To test whether p130Cas was essential for Gas6/Axl signaling activation, p130Cas was transiently silenced in SKOV3 cells. The cells were then serum starved, and stimulated or not with Gas6 during adhesion on FN. The p130Cas-silenced SKOV3 cells, adherent on plastic or on FN, showed a decreased level of phosphorylated Axl compared to the control-silenced cells (Fig. 3D). As expected, both unstimulated and Gas6-stimulated FN adherent cells showed higher levels of Axl phosphorylation with respect to the cells adherent on plastic.

Altogether, these data suggest that Gas6/Axl signaling leads to PI3K/AKT/rac activation in ECM-adherent ovarian cancer cells in a src-independent way. Furthermore, p130Cas is necessary for maximum Gas6-stimulated Axl activation.

### Reduction of Gas6-dependent adhesion and invasion following the impairment of p130Cas/Axl interactions

**Figure 4:**
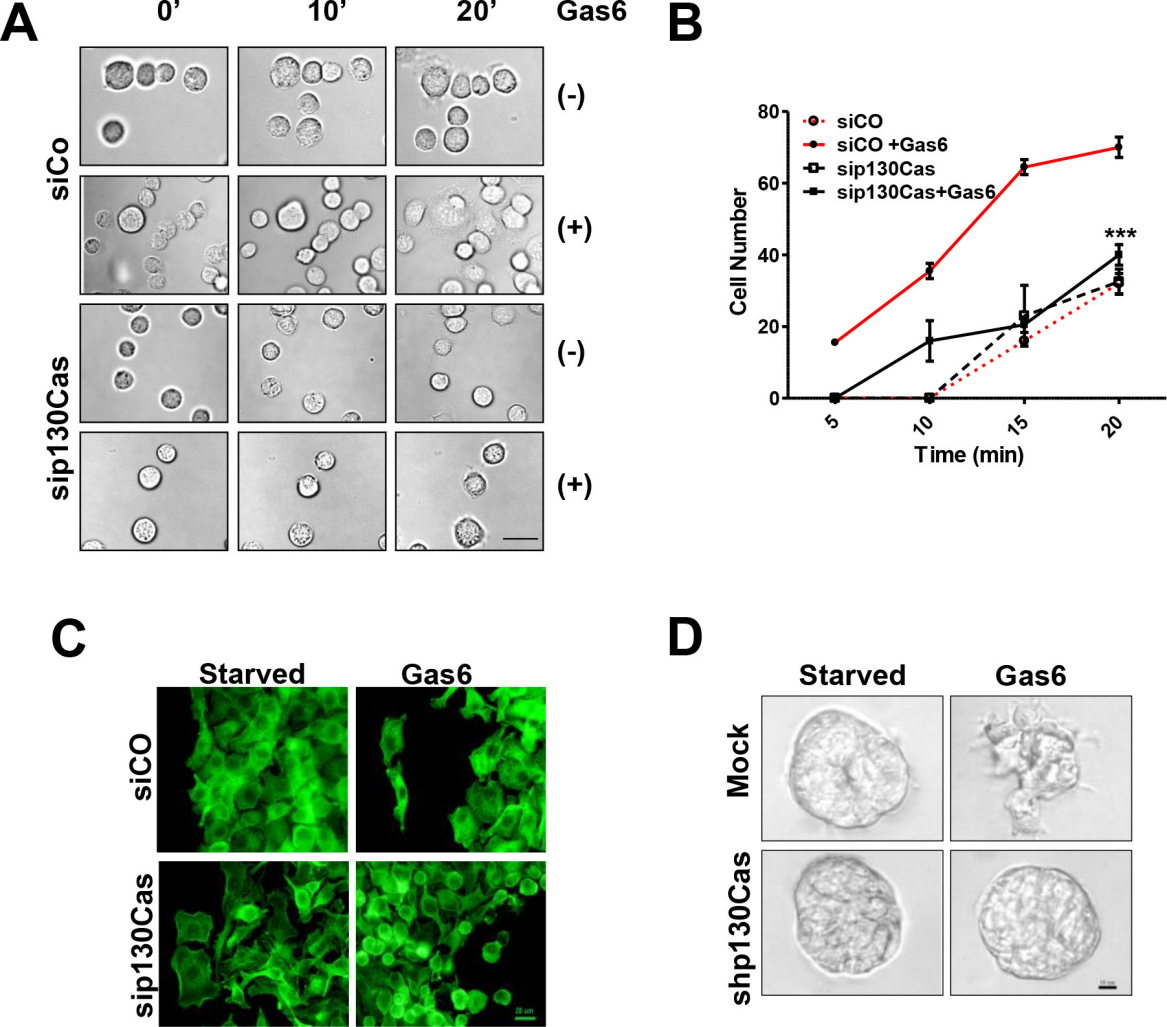
Reduction of Gas6-dependent adhesion and invasion following the impairment of p130Cas/Axl interactions **A.** Live cell imaging performed on untreated (−) or Gas6-stimulated control siRNA (siCO)- or p130 siRNA (sip130Cas)-transfected SKOV3 cells during adhesion on FN (see Supplementary Video 1 and 2, and Methods). Representative frames at 0, 10 min, and 20 min are reported. Scale bar, 100 μm. **B.** Graph reporting the number of FN adherent siCo- or sip130Cas-transfected SKOV3 cells, from the live imaging experiment, taken at different time points. Asteriscs indicate the significant differences between the two curves (paired *t* test, *p* = 0.0004) **C.** IF performed on cells as above after 20 min of adhesion on FN. The F-actin was stained with phalloidin (green). **D.** Invasion assay performed in Matrigel on starved or Gas6-stimulated SKOV3 cells stably p130Cas-silenced (shp130Cas) or infected with an empty shRNA vector (Mock). Representative images of the three independent experiments are shown.

To further clarify the role of p130Cas in the Gas6-dependent Axl activation and its contribution to ovarian cancer cell interaction with the ECM, SKOV3 cells were transiently transfected with siRNA targeting p130Cas or with a control siRNA, Silenced cells were then starved, and, while plated on FN, cell adhesion was monitored for up to 1 h by live imaging in the presence or absence of Gas6. Both the control and p130Cas-silenced SKOV3 cells did not adhere in the first 10 min and required 20 min to adhere to FN (Fig. 4A and 4B, and Supplementary Video 1). Upon Gas6 stimulation, the control-silenced cells started to adhere to FN after 5 min, and after 20 min were completely spread on the substrate (Fig. 4A and 4B and Supplementary Video 1 and 2), while a lower number of Gas6-stimulated p130Cas-silenced cells adhered on FN after 20 min (Fig. 4B) since the adhesion was impaired. Indeed, p130Cas-silenced cells, under Gas6 stimulation, formed an adhesion structure similar to membrane blebs emerging on the cell surface, but they were not able to complete the adhesion process (Supplementary Video 2).

To investigate the role of p130Cas in Gas6-dependent filopodia formation, actin cytoskeleton analysis was performed on control- and p130Cas-silenced SKOV3 cells plated on FN and stimulated with Gas6 (Fig. 4C). Upon Gas6 stimulation, control-silenced cells plated on FN showed the formation of a filopodia-like structure, as already observed in Fig. 3A, while p130Cas-silenced cells appeared rounded with bleb-like structures (as observed in the phase contrast microscopy image of Fig. 4A) and not attached to the substrate (Fig. 4C).

We next aimed to clarify whether p130Cas was also essential in the Gas6-dependent invasion. For this aim, an invasion assay was performed with SKOV3 cells stably silenced for p130Cas, using a shRNA-containing lentiviral vector, in order to follow the experiment for the required time. The infected Mock- and shp130Cas-SKOV3 cells, respectively, were embedded in reduced growth factor Matrigel and the obtained spheroids were then stimulated or not with Gas6. Upon Gas6 stimulation, the shp130Cas-SKOV3 cells maintained a perfect spherical shape (Fig. 4D), while the Mock-SKOV3 spheroids formed structures that invade into the surrounding Matrigel (Fig. 4D).

These data strongly indicate that Gas6-dependent adhesion mediated by integrin signaling/p130Cas is essential for adhesion to ECM and for invasion.

### Validation on ascites, patient-derived tumor cells, and on the biopsies of HGEOCs

**Figure 5:**
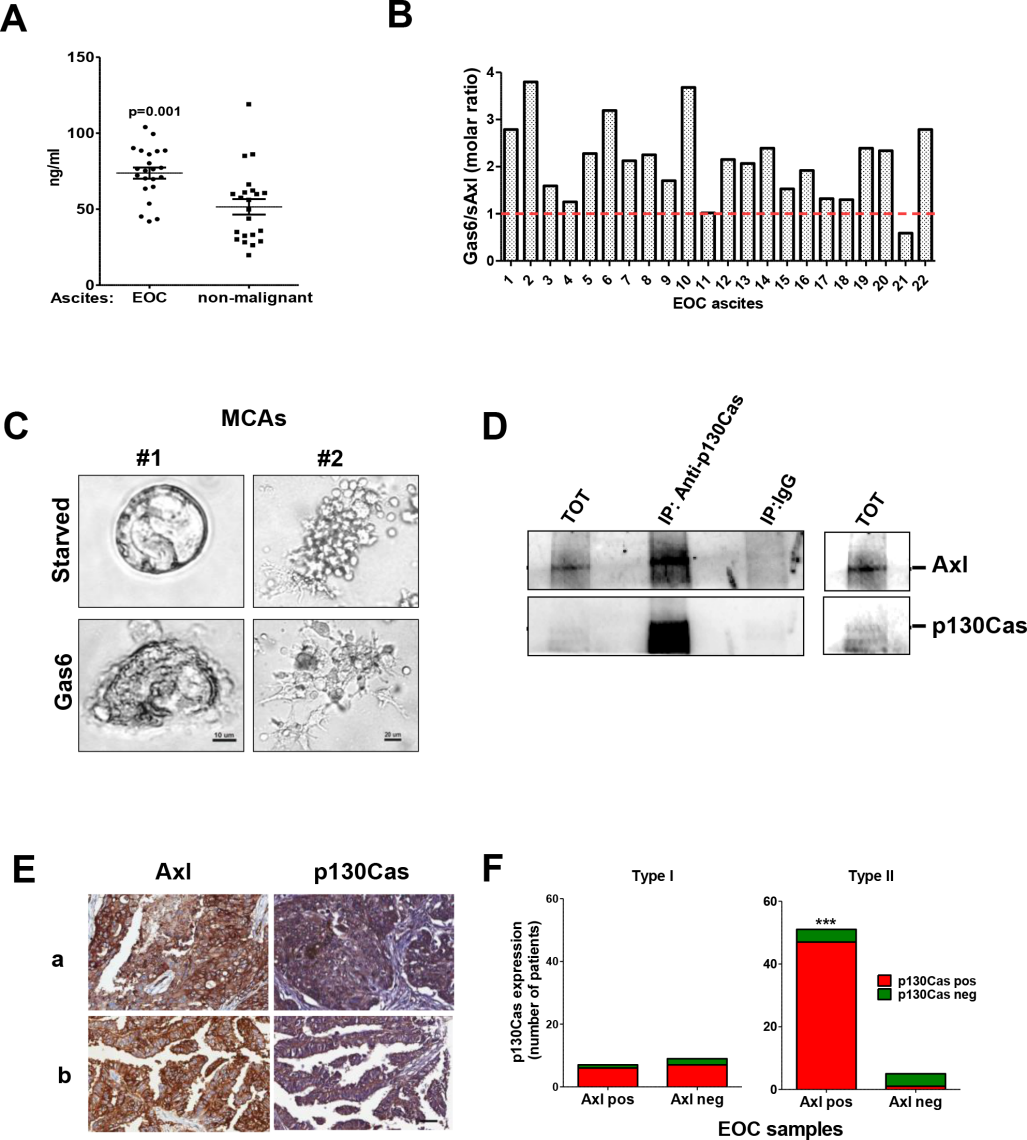
*In vivo* validation on HGEOC samples **A.** Gas6 levels measured by ELISA on 22 EOC ascites and 22 non-malignant ascites. **B.** Evaluation in the EOC ascites of the sequestration by the Axl ectodomain of Gas6. The dosage of the two molecules was performed as described in Methods. The graph reports the molar ratio between Gas6 and Axl ectodomain (sAxl). The red line represents the equimolar ratio. **C.** Invasion assay performed on starved or Gas6-stimulated MCAs from two representative Axl-positive MCAs from EOC ascites grown in Matrigel. EOC samples containing 80% of HGEOC cells, as evaluated by the cytopathologist, were processed. Satellite cells present in the starved sample are likely to be some immune cells present in the sample **D.** IP performed on the total cell lysates obtained from the EOC cells of patient #1 with anti-p130Cas. Immunoprecipitated samples were analyzed by western blotting with Abs against the proteins reported on the right. A longer exposure of the immunoblot with both Abs on the total lysates is shown on the right. **E.** Representative staining with anti-Axl and anti-p130Cas Abs on FFPE sections from two (*a* and *b*) HGEOC patients. Scale bar, 50 μm. **F.** Graph reporting the correlation between the p130Cas and Axl expressions in Type I and Type II (HGEOCs) tumors. The results of the contingency analysis are reported (Fisher's exact test, *p* = 0,0009).

To clinically validate the results obtained *in vitro* in established ovarian cancer cell lines, we first assessed the presence of Gas6 in 22 HGEOC ascites, whose characteristics have been described elsewhere [20]. We found that the levels of Gas6 ranged from 43 to 103 ng/ml, with a mean value of 74.1 ng/ml, and were statistically higher than the Gas6 levels in 22 ascites from non-malignant pathologies (Fig. 5A). To evaluate the possible sequestration of Gas6 by the Axl ectodomain present in the EOC ascites, the levels of this form of soluble Axl ectodomain was also measured. As shown in Fig. 5B, the Axl molecules were not able to completely sequester the Gas6 molecules in 20 out of the 22 ascites, indicating that Gas6 was available for the stimulation of Axl receptors expressed by HGEOC cells.

To test if Gas6 stimulation could exert the same effect on ovarian cancer cells obtained from malignant ascites, two samples from patients with serous HGEOC were allowed to adhere on Matrigel in the presence or in absence of Gas6 and the cancer cells invasion was monitored by phase contrast microscopy. In accord with what is observed for ovarian cancer cell lines, in HGEOC patient-derived spheroids, Gas6 induced a stellate invasive phenotype (Fig. 5C). IP with anti-Axl Ab performed on the total cell lysates obtained from the serous HGEOC of patient #1 demonstrated the association between Axl and p130Cas also *in vivo* (Fig. 5D).

In addition, the expression of Axl and p130Cas was analyzed by immunohistochemistry (IHC) on archival case material, which showed that, among 72 EOC primary tumors, the majority express Axl at the cell membrane (80%) (Supplementary Table 1). Eighty percent of the same EOC samples also co-expressed p130Cas in the cytoplasm (representative samples in Fig. 5E), and, moreover, Axl and p130Cas co-expression could distinguish between type I and type II (HGEOCs) as evaluated by Fisher's Exact Test (*p* = 0,0009, Fig. 5F).

These data, together with the biochemical and cellular results obtained *in vitro*, strongly support the idea that, in HGEOCs, the Gas6/Axl signaling cascade is activated in p130Cas-expressing cells.

### Identification of an Axl–driven signature in serous HGEOCs

We then analyzed Axl-expressing HGEOCs in order to assess their biological characteristics. Pearson's correlation analysis was performed to identify genes whose expression was significantly correlated with Axl expression in three data sets [4, 8, 21]. These data sets, reporting the gene expression profiles of a total of 976 HGEOC patients (Supplementary Table 2), allowed us to identify distinct HGEOC subtypes displaying different biological features with statistical prognostic relevance. Probes were filtered for an AXL-correlation higher than 0.4 and a *p* value lower than 0.05, and unique gene symbols were considered for further analysis. A comparison performed across the three data sets pointed out 121 concordantly correlated genes including Axl, (see the Venn diagram of Supplementary Fig. 4, Table 1 and Supplementary Table 3). These genes were enriched for those encoding ECM components (like collagens, FBN, FN, DCN) or adhesion molecules (like CDH11, and different integrin components); for those encoding matrix metalloproteases (MMP2 and 19, TIMP3), for epithelial-mesenchymal-transcription (EMT)-associated transcription factors (ZEB1, ZEB2); and for TGBB1, a classical EMT regulator. On the other hand, no significant correlation was found between Axl and the companion receptors, or its ligand Gas6 or the gene encoding p130Cas (BCAR1).

**Table 1:** Biological identification of the Axl-associated gene set

Biological Function^a^	Numbers of genes	Gene Symbol
**Actin cytoskeleton**	12	***ACTA2***, *ARHGAP15, ARHGEF6, CALD1*, ***DOCK10***, *DOCK2*, ***MARCKS***, ***PALLD***, ***PDLIM3***, ***PDPN***, *SAMSN1, TPM1*
**Adhesion molecules**	7	*CD84*, ***CDH11***, ***EMP3***, *EPB41L3, ITGA4, PECAM1*, ***TGFBI***
**ECM components**	13	***COL10A1, COL1A1, COL5A1, COL5A2, CRISPLD2, DCN***, *ECM2*, ***FBN1, FN1***, *IFFO1, MXRA8, OLFML2B*, ***OLFML3***
**Immune response**	26	*VSIG4, AIF1, ALOX5AP, C1QA, C1QB*, ***CD14***, *CD53, CD93*, ***COLEC12, CSF1R, CXCL12, CYBB, FCGR2B, FCGR3B***, *GIMAP6, HCK*, ***IL10RA***, *LAIR1, LAT2, LCP2, MSR1*, ***PTGER4***, *PTGIS*, ***SH2B3***, *SIRPA*, ***TLR7***
**Membrane receptors**	13	***AXL, EDNRA***, *EVI2A*, ***EVI2B***, *FZD1*, ***GPR65***, *HEG1, LAPTM5, MS4A4A, MS4A6A, PLXDC1*, ***PLXNC1***, ***TGFR2***
**Membrane trafficking**	8	*ATP6V1B2, ATP8B4*, ***COPZ2***, *DAB2, MARCH1*, ***PTPRC***, *PTRF, RAB31*
**Proteases**	10	***ADAM 12***, *CTSK, FAP, MMP19*, ***MMP2***, ***PCOLCE***, ***PLAU***, *RECK, SERPINF1*, ***TIMP3***
**Secreted molecules**	5	***ANGPTL2, FSTL1, HEPH, SFRP4, TGFB1***
**Transcription factors**	11	***AEBP1***, *FLI1, MAF, MAFB*, ***MEF2C***, ***MNDA***, *SNAI2, TGFB1/1, ZCCHC24, ZEB1*, ***ZEB2***
**Others**	16	*CSGALNACT2*, ***ENTPD1***, *GLIPR1, GLT8D2, LHFP*, ***LHFPL2***, *MFSD1*, ***NPL***, *PKD2*, ***PLEK***, ***PMP22***, ***QKI***, ***RGS4***, ***RNASE6***, *SPATS2L, TM6SF1*

a Genes are grouped according to their function. Genes included in the Axl-driven signature are in bold.

To validate these data on clinical HGEOC samples, real-time RT-PCR was performed on RNA extracted from eight serous HGEOCs (Supplementary Table 4) for eight genes (CDH11, CXCL12, DCN, FN1, MMP2, PLAU, TGFB1, ZEB2) included in the identified 121-gene set. Among these genes, the expression of four of them (DCN, FN1, MMP2, and PLAU) was significantly correlated to AXL expression (R^2^ range: 0.76–0.86; *p* value range: 0.0048–0.00012), while that of CDH11 showed an R^2^ = 0.48 but a borderline significance (*p = 0.057*), and the expression of three of them (CXCL12, TGFB1, and ZEB2) was not correlated to AXL (Supplementary Fig. 5).

To provide an insight into the possible biological significance of the 121-gene set, a functional analysis of the correlated genes was carried out by Ingenuity Pathway Analysis software (IPA) [22]. Among the five top Molecular and Cellular Functions, ‘Cellular movement’ and ‘Cellular morphology’ were included (Supplementary Table 5A). Two of the five networks associated with the highest score, named N1 and N2, were ‘Connective Tissue Disorders, Dermatological Diseases and Conditions, Developmental Disorder’ and ‘Cancer, Organismal Injury and Abnormalities, Reproductive System Disease’ (Supplementary Fig. 6). N1 contained several ECM proteins (different types of collagen, CDH11, DCN), together with the integrin αvβ3 receptor genes, and AKT as the hub gene. N2 included genes associated to cell morphology and to the actin cytoskeleton (Supplementary Table 5B). These data, obtained on a large number of HGEOC patients, are concordant with the *in vitro* data of Gas6/Axl-induced AKT activation and of a crosstalk between the Axl and ECM/integrin signaling pathways.

**Figure 6:**
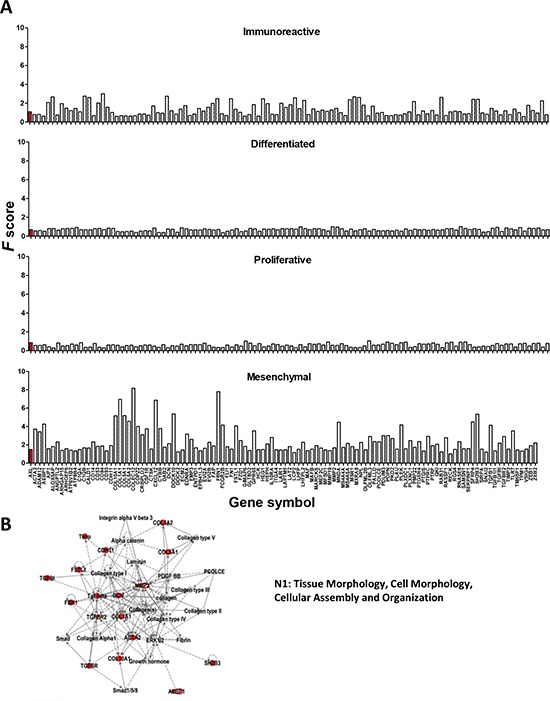
A. Identification of an Axl–driven signature in serous HGEOCs **A.** Evaluation of the expression changes of the genes included in the Axl-associated gene set in each of the HGEOC subtypes according to the TCGA data set [5]. A total of 114 out of the 121 genes identified were plotted, due to differences in the gene symbols. Only the genes reported by Verhaak *et al*. [5] in the Supplementary Table 8A are plotted. The bars corresponding to Axl are reported in red. **B.** Graphical representation of the network identified by IPA. The top five networks, reported in Supplementary Table 5B, were identified by loading the genes reported in bold in Table 1. The Axl-correlated genes included in the network are highlighted in red.

To investigate whether the Axl-associated gene set could impact on a specific HGEOC subtype, the change in expression of each gene of our gene set was evaluated for each subtype identified in data set II [5]. The entire Axl-associated gene set increased in the ‘mesenchymal’ subtype while it was decreased in the ‘proliferative’ subtype and in the ‘differentiated’ subtype (Fig. 6A), strongly suggesting that the tumors from these subtypes display different altered signaling pathways. On the other hand, the ‘immunoreactive’ subtype presented both increased and decreased genes (Fig. 6A). In the ‘immunoreactive’ subtype, the increase of Axl was lower than in the mesenchymal subtype (gene rank *F* score = 1.6 vs 8.6, as evaluated by Verhaak *et al*. [5]). This was likely due to the presence of a large number of immune cells in these samples, as also stated by the same authors [5]. Hence, to select for Axl-correlated genes likely altered only in HGEOC cells, all genes showing an *F* score ≤ 1.6 were excluded from the Axl-associated gene set (see the Methods section), thus generating an Axl-driven signature of 62 genes. Seventy percent of the ECM molecules and 100% of the secreted molecules were retained by applying this cut-off, whereas 50% or less of the molecules belonging to other categories, including to the immune response, were retained. IPA analysis performed on this gene list identified five top networks, of which the highest score was observed with ‘Tissue Morphology, Cell Morphology, Cellular Assembly and Organization’ (Fig. 6B). Besides the molecules of the Axl-driven signature (reported in red in Fig. 6B), this network also included the ECM component ‘laminin’, and also ‘integrin alpha V beta 3′, whose functionality is necessary for Gas6/Axl activation (see Fig. 2D). Overall, these findings further support the *in vitro* data presented above.

### Axl-driven signature identifies the HGEOC patients with poor overall survival

**Figure 7:**
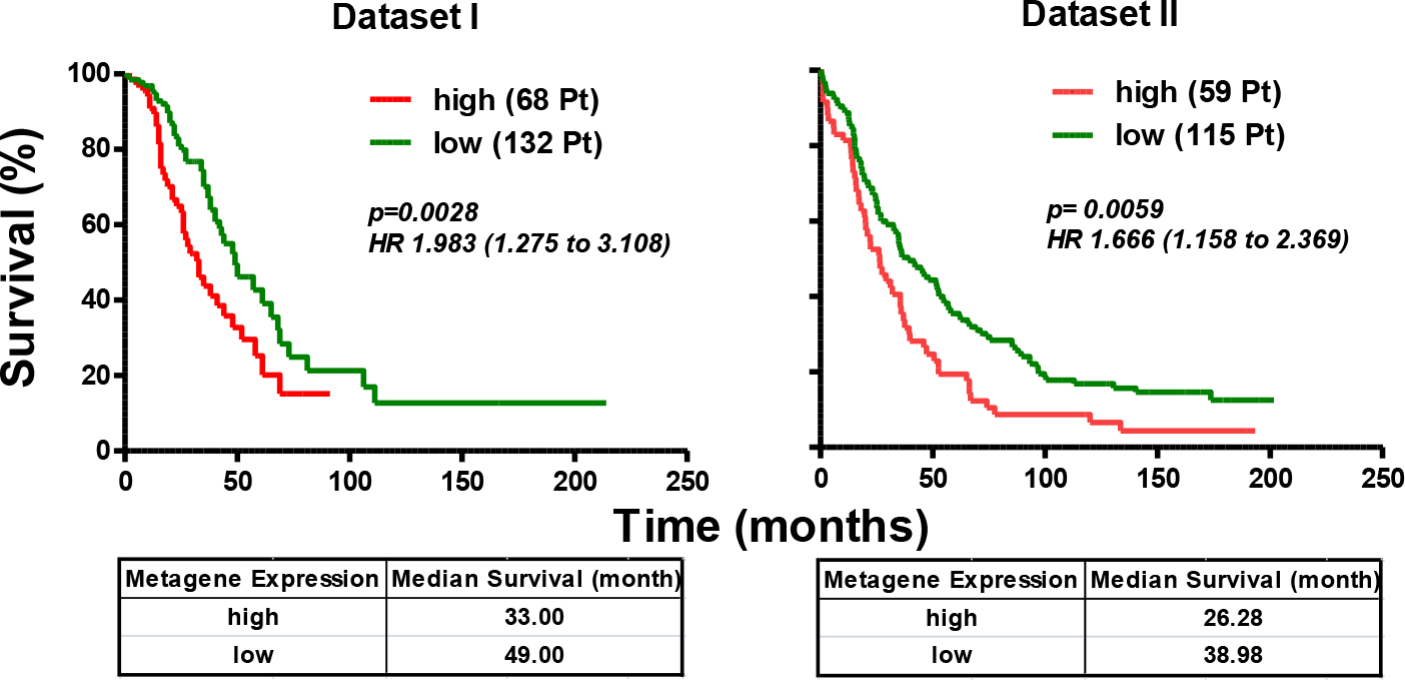
Axl-driven signature identifies the HGEOC patients with poor overall survival Kaplan-Meyer curves, log-rank *p* values and HR are used to compare OS between HGEOC patients with ‘high’ (red lines) and ‘low’ (green lines) expression intensities of the metagene consisting of the Axl-driven signature. The tables below each curve reports the median survival of each group of patients.

To investigate the clinical relevance of this newly identified Axl-driven signature, we analyzed the correlation of AXL-driven signature expression with overall survival (OS) in data sets I and II by comparing the survival rates in patient groups with a ‘high’ and ‘low’ expression of the metagene consisting of the Axl-driven signature. In each of the two data sets analyzed, the patient group with a ‘high’ expression of the metagene had poor OS (log-rank *p* values of 0.0028 and 0.0059, respectively, Fig. 7). For data set I the adjusted hazard ratio (HR) was 1.983 (95% confidence interval (CI) = 1.265–3.108), and for data set II, the HR was 1.666 [95% CI = 1.158–2.396]. Furthermore, HGEOC patients of data set I with ‘high’ metagene expression relapsed earlier than those with ‘low’ metagene expression (Supplementary Fig. 7).

Overall, our data establish for the first time a crosstalk between the Axl and ECM/integrin/p130Cas signaling pathways and identify an Axl-driven gene signature that could possibly be useful for the identification of HGEOC patients with poor outcome.

## DISCUSSION

In this report, we unravel a novel signaling cascade induced upon Gas6 stimulation of HGEOC cells, whereby the Gas6/Axl axis, through a crosstalk with the integrin adaptor p130Cas, induces the invasion in Matrigel of ovarian cancer spheroids from cell lines and HGEOC patient-derived cells. In *in vivo* validation, represented by HGEOC samples, the Gas6 concentration was found to be elevated in HGEOC ascites, and Axl and p130Cas were co-expressed in type II HGEOCs. Clinically, we identified an Axl-driven molecular signature, whose predicted biological function basically recapitulated the signaling observed *in vitro*, and we were thus able to identify the HGEOCs with the poorest outcome.

In the last few years, Axl has been extensively studied and is described as a determinant of metastasis formation and progression for various types of solid tumors, including EOCs (for review see [10, 15]). However, this is the first report that deeply dissects the cellular and molecular events occurring in the EOC cells upon Axl activation due to Gas6 stimulation. The novel finding that the Gas6/Axl signaling cascade cooperates with the integrin pathway through p130Cas for the adhesion and invasion of EOC cells suggests that the process of EOC dissemination involves a plethora of different stimuli inducing the activation of more than a single signaling pathway. In a model of renal cell carcinoma, Gas6/Axl signaling induces metastasis formation through a crosstalk with src kinase and RTK Met [23], indicating that the molecular requirements necessary for Axl activation may be tumor-specific. Under physiological conditions, the crosstalk between integrins and the Axl receptor has so far only been observed in endothelial cells, and in this model, Axl tyrosine phosphorylation was induced in a Gas6-independent fashion by the formation of a complex between Axl and the β3 integrin, leading to endothelial cells survival [24]. Accordingly, we found that the inhibition of the interaction between the β3 integrin and the ECM led to a decrease in Axl phosphorylation; however, in contrast with the results on endothelial cells, in HGEOCs, the activation of Axl was dependent on both Gas6 stimulation and β3 integrin engagement.

The convergence between Axl and integrins was mediated by the adaptor protein p130Cas. P130Cas is a scaffold protein that is involved in the process of cell adhesion and migration upon integrin engagements with the ECM [25]. Although no correlation was observed between Axl and BCAR1, the gene encoding p130Cas, a significant association was observed between the Axl and p130Cas proteins with type II HGEOCs. This discrepancy is probably due to the different stability of the p130Cas transcript compared to the protein. In line with these results, high p130Cas expression was previously found in EOC and was associated with the advanced tumor stage, and hence decreased progression-free survival or overall survival [26]. Furthermore, p130Cas silencing in EOC cells induces an *in vivo* alteration of the apoptosome and increases cell death upon etoposide treatment. Moreover, the role of p130Cas in regulating tumor cell invasion through the release of matrix metalloproteinases such as MMP-9 has also been reported [27]. In colon cancer, the increased expression and activity of Src tyrosine kinase has been associated with persistent activation of the p130Cas/JNK pathways, the induction of MMP-2 and MMP-9, and invasion/survival in an α1-integrin-dependent fashion [28]. In several cancer cell lines, the association of the FAK–p130Cas complex to the focal adhesion sites has been described as being required for MMP-induced matrix degradation and cell invasion [29]. In EOC, Axl activation has been linked with cell invasion through the induction of MMP-1 and MMP-9 [11]. Accordingly, we show here that the MMP-2 gene is included in Axl-associated signature, suggesting that the final consequence of the Axl and p130Cas crosstalk is the production of MMPs, which in turn leads to the invasion of EOC cells.

We demonstrated that during the process of cell invasion, the interaction between EOC cells and the ECM activates Gas6/Axl signaling cascade which in turn triggers the invasion process through the rac activation and actin remodeling. Very recently, a rac1/Pak1/p38/MMP-2 axis has been found to contribute to angiogenesis and the invasion of EOCs [30]. In breast cancer cells, Gas6/Axl signaling also leads to rac activation and invasion through the scaffold protein Elmo and the RHO-GTPase activator DOCK1 [31]. Our computational analysis highlighted DOCK10 within Axl-co-regulated genes, further indicating the importance of the actin remodeling for the Axl-expressing HGEOCs. The association between Axl activation and cytoskeleton organization was also supported by IPA analysis on the Axl-driven signature of 62 genes, in which the identified network with the highest score was indeed associated with cell morphology. For all these reasons, investigations are ongoing to clarify the role of the DOCK activators and actin binding proteins during EOC progression.

The process of HGEOC peritoneal dissemination requires the re-attachment of EOC MCAs to the peritoneum or omentum, thus generating secondary lesions; this mechanism has been called mesothelial cell clearance [32]. Recently, a mesenchymal reprogramming of HGEOC MCAs was observed during mesothelial clearance [33]. In this report, Axl was one of the proteins whose expression increased in HGEOC cells competent for the mesothelial cell clearance. The requirement of adhesion to ECM for maximal Axl activation shown here is in accord with the notion that Axl could be a determinant of mesothelial cell clearance. The presence of Gas6 in HGEOC ascites and in the conditioned media of several EOC cell lines suggests that Gas6, released from the EOCs MCAs or by the cells of the immune system, acts like a fuel to favor tumor invasion and the establishment of secondary lesions.

Global gene expression analyses from the three data sets used in the present study help us identify novel molecular subtypes of HGEOCs based on their peculiar molecular signature and associated distinct biology [4, 5, 8]. Our data showed that the transcripts of Axl and that of co-regulated genes are increased in the subtype called ‘stroma’ by Tothill *et al.* [4], ‘mesenchymal’ by Verhaak *et al*. [5], and ‘mesenchymal-like’ by Konecny *et al*. [8]. Besides the denomination, this subtype is characterized by increase of the transcripts of genes encoding for EMT and ECM molecules, and in all the studies, it displays a significant shorter overall survival. In addition, bioinformatics tools indicated TGFB1 to be an up-stream regulator of the Axl-associated signature, which includes several ECM proteins. A collagen-remodeling gene signature, regulated by TGF-β signaling, has also been found to be associated with metastasis formation and a poor prognosis [34]. Although this signature only included two genes, COL5A1 and TIMP3, which are common to the Axl-associated signature, together these data indicate that the most aggressive HGEOCs display peculiar extracellular components whose expression should be taken into account when alternative therapeutic approaches are considered for these patients.

Interestingly, the genes increased in the above-mentioned HGEOC subgroups were decreased in the HGEOC subtype named ‘proliferative’ [4, 21] and ‘proliferative-like’ [8], which are characterized by a slightly better outcome. These data clearly indicate that the biology of HGEOCs included in the mesenchymal subgroup is different from the biology of HGEOCs belonging to the proliferative subtype. We can hypothesize that the signaling pathway identified in this study is one of the pathways activated in the ‘mesenchymal’ HGEOC subtype. Our experimental data represent a step forward in gaining an understanding of the molecular mechanism associated with the progression of HGEOCs and suggest that the Gas6/Axl/p130Cas axis can be exploited as a therapeutic target in combination with chemotherapy to cure those patients whose tumors molecularly recapitulate the most aggressive EOC subtype, which will likely recur earlier with the formation of new metastases and uncontrolled dissemination.

HGEOC is a clinically separate and molecularly heterogeneous disease comprising subtypes with different response rates to conventional chemotherapy. The definition of a molecular signature for the identification of HGEOC patients, as well as for one of the pathways possibly activated in these HGEOCs, may help to provide a more effective and tailored therapeutic approach.

## MATERIALS AND METHODS

### Antibodies and reagents

Abs against P-Axl(Y702) (rabbit), P-AKT(S473) (rabbit), anti-Mer (mouse), P-src (Y416), and anti-P-Tyrosine (P-Tyr-100) (mouse) were obtained from Cell Signaling Technology (New England BioLabs, Beverly, MA, USA); anti-Axl (goat), anti-FAK (rabbit), anti-Tyro-3 (goat), and anti-β1 and anti-p130Cas (mouse) from Santa Cruz Biotechnology (Santa Cruz, CA, USA); anti-α-tubulin (mouse) from Thermo Scientific (Fremont, CA, USA); anti-β-actin (rabbit) from Sigma-Aldrich (Saint Louis, MO, USA); anti-β3 from Abcam (Cambridge, UK); and anti-β4 from Millipore (Merck Millipore, Oxford, UK).

Alexa Fluor 488 phalloidin was obtained from Molecular Probes (Invitrogen, Carlsbad, CA, USA). Human recombinant Gas6 and Axl-Fc were from R&D systems, Inc. (Minneapolis, MN, USA), as was the ELISA for Gas6 dosage. FN was from Sigma-Aldrich. The Taqman Gene Expression Assays were from Applied Biosystems (Foster City, CA USA). Puromicine and Lipofectamine 2000 were from Invitrogen. R428, the Axl inhibitor, was from Rigel (South San Francisco, CA, USA); EHT1864, the Rac inhibitor, was from Tocris (Minneapolis, MN, USA).

### Cell lines

The human ovarian cancer cell lines used in this study were: IGROV-1, kindly provided by Dr Bénard (Paris, France) [35]; SKOV3, from ATCC; OVCAR4 and OVCAR5, provided by Dr Camalier (NCI-NIH, USA); and NL3507, from Dr van den Berg-Bakker (Leiden, the Netherlands) [36]. They were maintained in RPMI 1640 medium (Sigma-Aldrich) supplemented with 10% fetal calf serum (FCS) (Hyclone, Logan, UT, USA) and 1% L-glutamine at 37°C in a humidified atmosphere of 5% CO_2_ in air. OAW42 was provided by Dr A. Ullrich, (Max Planck Institute of Biochemistry, Martinsried, Germany) [37], and maintained in DMEM supplemented with 10% FCS and 1% L-glutamine in a humidified atmosphere of 5% CO_2_. Cells were genotyped at the Functional Genomic facility of our Institute, using a Stem Elite ID System (Promega, Madison, WI, USA), according to the manufacturer's instructions and ATCC guidelines. Cells were routinely confirmed to be mycoplasma-free by Hoechst staining and by using a MycoAlert Mycoplasma Detection Kit (Lonza, Basel, Switzerland).

For the biochemical analysis, Gas6 stimulation was performed on starved cells in serum-free medium for 30 min at a concentration of 500 ng/ml. Recombinant human Axl-Fc chimera was used at 2.5 μg/ml and added 1 hr before the addition of Gas6 or the serum-free medium.

### Patients and samples

Twenty-two ascites from EOC patients, whose characteristics are reported elsewhere [20], were collected at the time of debulking surgery. The Institutional Review Board approved the use of archived material and ascites, as well as the clinical data. All the clinical specimens were accompanied by the informed consent from all patients to use the excess biological material for investigative purposes. The histological selection of patients was based on them being at an advanced stage at diagnosis and the presence of ascites at surgery. Samples of nonmalignant ascites were obtained from cirrhotic patients that had undergone therapeutic paracentesis and had been admitted to the Clinica Medica Ward of AOU Maggiore della Carita’ University Hospital at Novara, Italy. Patient-derived 3D cultures were obtained by using MCAs from serous HGEOC ascites collected at the time of surgery.

The study group consisted of 72 cases of EOCs diagnosed between January 1, 2006 and December 31, 2011 in “St. Spiridon” Emergency Clinical Hospital and “Cuza Vodă” Obstetrics and Gynecology Clinical Hospital from Iassy, Romania. The study was approved by the Ethics Committee of the “Grigore T. Popa” University of Medicine and Pharmacy, Iassy, based on the patients' informed written consent for the usage of the biologic material. The TMA preparation and the immunohistochemical investigation were performed at Fondazione IRCCS Istituto Nazionale dei Tumori, Milan, Italy.

### RNA extraction and real-time RT–PCR analysis

Total RNA from ovarian cancer cell lines was extracted using a commercial kit (Amersham Bioscience-GE Healthcare, Piscataway, NJ, USA). Frozen tissues were homogenized using a bead-mill device (TissueLyser, QIAGEN Sciences, Germantown, MD, USA), then total RNA was purified with the NucleoSpin miRNA (Macherey-Nagel, Duren, Germany) according to the manufacturer's instructions. For the formalin-fixed, paraffin-embedded (FFPE) HGEOC samples, RNA was isolated with the miRNeasy FFPE Kit (QIAGEN Sciences), following the manufacturer's instructions. A High-Capacity cDNA Reverse Transcription Kit (Applied Biosystems) was used to perform the reverse transcription reaction according to the manufacturer's instructions. Real-time RT-PCR was performed using the 7900 system (Applied Biosystems) TaqMan Fast Universal PCR Master Mix (Applied Biosystems), according to the manufacturer's instructions. TaqMan Gene expression assays (Applied Biosystems) were used and are listed in the Supplementary Table 6.

### Western blotting and IP

Cells were washed with ice-cold PBS containing sodium ortovanadate (0.1 mM) and lysed with NuPAGE^®^ LDS Sample Buffer (1x) (Invitrogen) plus β-Mercapto. Proteins were separated on precast 4–12% SDS-polyacrylamide gels (Invitrogen). A western blot was performed as described [20] and analyzed using a ChemiDoc XRS system and the Quantity One^®^ software from Biorad (Hercules, CA, USA).

IP was performed, essentially as described [38]. Briefly, cells were lysed in cold lysis buffer (150 mM NaCl, 50 mM Tris-HCl, pH 7.4, 0.25% NaDoc, 1 mM EDTA pH 8, 1 mM Na_3_VO_4_, 1% PMSF, 1% Np-40, and protease inhibitors) for 30 min on ice. The cellular debris and nuclei were removed by centrifugation at 13000 rpm for 30 min at 4°C. Normal rabbit or mouse sera were used as the negative control. Primary ab was bound to beads conjugated with goat anti-mouse or anti-rabbit ab (Dynabeads; Dynal ASA, Oslo, Norway) and incubated with cell extracts overnight at 4°C with rotation. Beads were washed once with cold lysis buffer, twice with PBS plus BSA, and with protease inhibitors (10 min/wash). Immunoprecitated proteins were separated by SDS-mini-gels (Invitrogen) under reducing conditions.

### 3D cultures and the invasion assay

In a 3D Algimatrix™ culture system 96-well plate (Gibco, Carlsbad, CA, USA), cells with a density of 1 × 10^3^ were incorporated into the 3D alginate scaffold in 100 μL of media, according to the manufacturer's protocol. After 20 min, another 150 μL of media was added and the cells were grown in the incubator at 37°C with 5% CO_2_. The media was changed every 5 days. Gas6 treatment was started at 12 days post cell seeding. In some experiments, the spheres were dissolved from the Algimatrix scaffold according to the manufacturer's protocol and seeded into Matrigel in a serum-free medium or in the presence of Gas6 500 ng/ml. Morphological changes in the spheres shapes were monitored for up to 48 hr using an inverted microscope with a 20X or 40X 0.75 NA PlanFluor objective (Nikon, Tokyo, Japan). The images were acquired with ACT-1 software (Nikon).

For the Matrigel 3D culture, cells (1 × 10^3^) were suspended in a medium of growth factor-reduced Matrigel (BD Biosciences, Bedford, MA, USA) and then seeded directly onto uncoated 48-well culture plates. The plates were first incubated for 30 min at 37°C and then the complete medium was added. Upon formation of the spheres (after about a week), an invasion assay was performed on cells starved overnight. Starved cells were stimulated with Gas6 (500 ng/ml). For the HGEOC patient-derived 3D culture, cells were re-suspended in serum-free medium alone or in the presence of 500 ng/ml Gas6 and plated onto growth factor-reduced Matrigel in 48-well plates. Drug pre-treatment for the invasion assay was performed upon formation of the spheres. Morphological changes of the spheres were monitored for up to 48 hr using an inverted microscope with a 20X or 40X 0.75 NA PlanFluor objective (Nikon). The images were acquired with ACT-1 software (Nikon). A medium number of 30 spheres was analyzed for each experiment.

### Live imaging

Serum-starved SKOV3 cells, transfected with siRNA against p130Cas or with a control siRNA for 48 hr, were left in suspension for 1 hr before being seeded on FN (20 ug/ml) pre-coated 8-well glass chamber slides (Nalge Nunc International, NY, USA), and were then stimulated or not with Gas6 500 ng/ml. Cell-adhesion on FN was monitored for up to 1 hr by live-imaging using a 20X 0.5 NA Plan-Fluor DIC dry objective. The microscope (NIKON TE300 ECLIPSE) was equipped with an incubation chamber, which provided a humidified atmosphere at 37°C with 5% CO_2_. Cells were counted based on five field digital images taken randomly. The average number of cells and SD were calculated based on triplicate experiments.

### IF

Cells seeded on 8-well glass chamber slides pre-coated with FN 20 μg/ml (Nalge Nunc International) were grown for 24 hr, serum starved and then stimulated or not with Gas6 500 ng/ml. Before immunostaining, the cells were fixed with 2% paraformaldehyde for 20 min and permeabilized for 10 min in PBS/Tween 0.1%. The samples were then blocked for 30 min with PBS/1% BSA. Samples were mounted with Prolong Gold Antifade reagent with DAPI (Invitrogen) and analyzed using an Eclipse TE2000-S microscope with a 40X PlanFluor objective (Nikon). Images were acquired with ACT-1 software (Nikon). All the procedures were carried out at room temperature.

### Flow cytometry

Cells were permeabilized in 70% cold ethanol for 30 min on ice and stained by sequential incubation with primary and secondary mAbs. Cells were analyzed for antigen expression by a FACSCalibur cytofluorimeter (BD Biosciences). The percentage of positive cells and the median fluorescence intensity after background subtraction were recorded.

### siRNA and transfection

Cells were transfected with 40 nmol/ml of small-interfering RNA (siRNA) duplex specific for p130Cas (Smart Pool, Thermo Scientific, Dharmacon, Inc. Chicago, IL, USA) or control siRNA (Quiagen). Transfection was performed using Lipofectamine 2000 (Invitrogen) according to the manufacturer's protocol. Whole cell lysates were prepared 48 hr after transfection. In one of the experiments, serum-starved p130Cas-silenced or control-transfected SKOV3 cells were left in suspension for 1 hr before being seeded on FN (20 ug/ml) and stimulated or not with Gas6 500 ng/ml for 30 min, and the total cell lysates were analyzed by western blotting.

### Stable p130Cas silencing

The pLKO.1 lentiviral vector carrying a shRNA directed to human p130Cas (p130Cas shRNA) was selected in the pLKO.1 target gene shRNA set (clone ID TRCN0000115985), purchased from Open Biosystem (Huntsville, AL, USA). The pLKO.1 scramble shRNA vector (Addgene, Cambridge, MA, USA) was used as the negative control. Lentiviral particles were generated and concentrated by ultracentrifugation (50,000 g, 2 hr). SKOV3 cells were infected with the lentiviral p130Cas-shRNA (sh-p130Cas) and scramble-shRNA (Mock). Puromycin (Sigma-Aldrich) (0.8 lg/ml) was added 24 hr after infection with the PLKO.1 vectors.

### Gas6 and soluble Axl assay

Gas6 was measured with a sandwich ELISA modified from that already published [13]. Briefly, a 96-well plate (ImmunoPlates MaxiSorp F96, NUNC, Hereford, UK) was coated overnight with anti-Gas6 primary antibody (goat polyclonal affinity purified IgG, R&D Systems). The antigen was detected by a secondary biotin-conjugated antibody (Biotinylated anti human Gas6 antibody, R&D Systems), a streptavidin-peroxidase conjugate (Sigma-Aldrich), and TMB (3,3′,5,5′-tetramethylbenzidine, Sigma-Aldrich). The reaction was blocked with sulphuric acid 1.8 M and the absorbance detected at 450 nm with the reference wavelength set at 570 nm. The optical density was fitted against the nominal concentration by applying a four-parameter logistic regression to the calibration curve prepared in BSA (bovine serum albumin, further purified fraction V, ≥98%, Sigma-Aldrich).

A soluble Axl assay was performed with a commercial ELISA kit (DuoSet IC R&D Systems), following the manufacturer's instructions.

### IHC

Paraffin-embedded EOC tumors were analyzed with anti-Axl and anti-p130Cas Abs. IHC was performed after deparaffinization, as described [38]. The primary goat anti-human Axl Ab was diluted 1:25 and the primary mouse anti-human p130Cas Ab was diluted 1:400. Incubation with each of the primary antibodies was performed overnight at 4°C, then the slides were incubated for 30 min at room temperature with the secondary biotinylated antibodies diluted 1:200. The slides were washed with PBS, and the peroxidase activity was revealed by incubating sections in DAB (3–3′diaminobenzidine) (DAKO, Denmark) for 5 min. After washing with water, sections were counterstained with Gill's hematoxylin solution for 5 sec.

### Computational analysis of the gene expression data

Gene expression data were downloaded as raw signals from GEO (GSE9891 [4]) and from the TCGA repository (https://tcga-data.nci.nih.gov/tcga) for data set I and II, respectively, or as a processed matrix from GEO for data set III (GSE53963 [8]). Raw gene expression data were RMA-normalized through the Expression Console software (Affymetrix, Santa Clara, CA, USA). Data set I was filtered for the serous histotype, for consistency with the two other data sets. The AXL probe 202686_s_at was selected for the two data sets processed through the Affymetrix platform (data sets I and II), while the probe A_23_P208389 was selected for the Agilent data set (data set III). Among each data set, the selected AXL probe was analyzed for Pearson's correlation against all the other probes by using the cor, cor.test, and p.adjust bioconductor functions in the R programming language (version 3.0.2, http://www.r-project.org), in order to calculate the Pearson's correlation coefficients (r), the *p* value, and the Benjamini and Hochberg FDR, respectively. The AXL-correlated probes were filtered for *r* ≥ 0.4 and FDR ≤ 4.53E-08. The Axl-associated gene set comprised the 121 genes commonly correlated in all of the three data sets. This Axl-associated gene set was then screened in the four HGEOC subtypes, as described by Verhaak *et al.* [5] in the Supplementary Table 8A (Fig. 6A), and only the genes up-regulated in the mesenchymal subtype with an *F* score higher than 1.6 were conserved. The resulting reduced list of 62 genes was thus considered the Axl-driven signature. The F score for a given gene is defined by Verhaak *et al.* [5] as the measure used by the significance analysis of a microarray to indicate the differences in gene expression between two groups.

The Axl-associated gene set of 121 genes and the Axl-driven signature were further analyzed by Ingenuity Pathway Analysis (IPA), and a core biological pathway analysis was performed to identify the molecular networks and upstream regulators.

### OS analysis of the microarray data

We performed the survival analysis using a single metagene from the Axl-driven signature of the 62 genes. We calculated a single metagene value for each data set by performing the mean of the expression values of the genes in each patient. Finally, in each data set, we divided patients with a metagene value higher than the second tertile from all the others and performed the survival analysis. The ‘risk index’ of the 62 genes was calculated from a linear combination of the gene expression values and their estimated multivariable Cox proportional hazard regression coefficients. The median risk index was used to define the two patient groups: one group was characterized by a high expression of the 10 genes and the other by a low expression of the 10 genes. The Kaplan–Meier method was used to estimate OS and the log-rank test was applied to compare OS across the groups. All the analyses were performed using R packages.

### Statistical analyses

GraphPad Prism software (GraphPad Software, San Diego, CA, USA) was used to analyze all the data. Differences between the mean values were analyzed by Student's *t* test and a two-way ANOVA. The correlation of Axl and p130Cas expressions in IHC was evaluated by Fisher's exact test. *P* values < 0.05 were considered significant.

## SUPPLEMENTARY FIGURES AND TABLES, VIDEOS


