# Supplementary material for: Novel Axl-driven signaling pathway and molecular signature characterize high-grade ovarian cancer patients with poor clinical outcome
Source: Oncotarget. 2015 Sep 4;6(31):30859–75. doi: 10.18632/oncotarget.5087 (PMC4741573; doi:10.18632/oncotarget.5087)
Supplement: Supplementary file 1 [file oncotarget-06-30859-s001.pdf]

SUPPLEMENTARY FIGURES AND TABLES

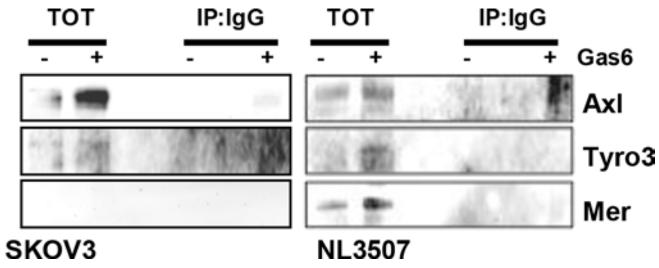

Supplementary Figure S1: IP performed on lysates from starved or Gas6-stimulated SKOV3 and NL3507 cells with Normal rabbit (IgG) as negative control of the IP reported in Figure 1C. Immunoprecipitated samples were analyzed by western blotting with Abs against the proteins reported on the right.

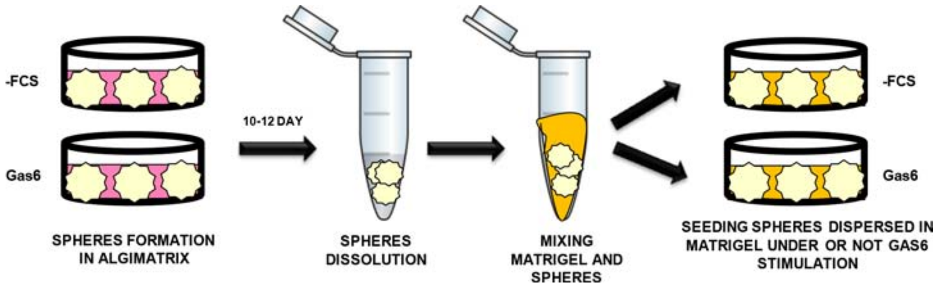

Supplementary Figure S2: Schematic representation of the experiment reported in Figure 2B. Cells were grown in Algimatrix™ in presence or not of Gas6 and then dissolved and passed in Matrigel® in the same conditions.

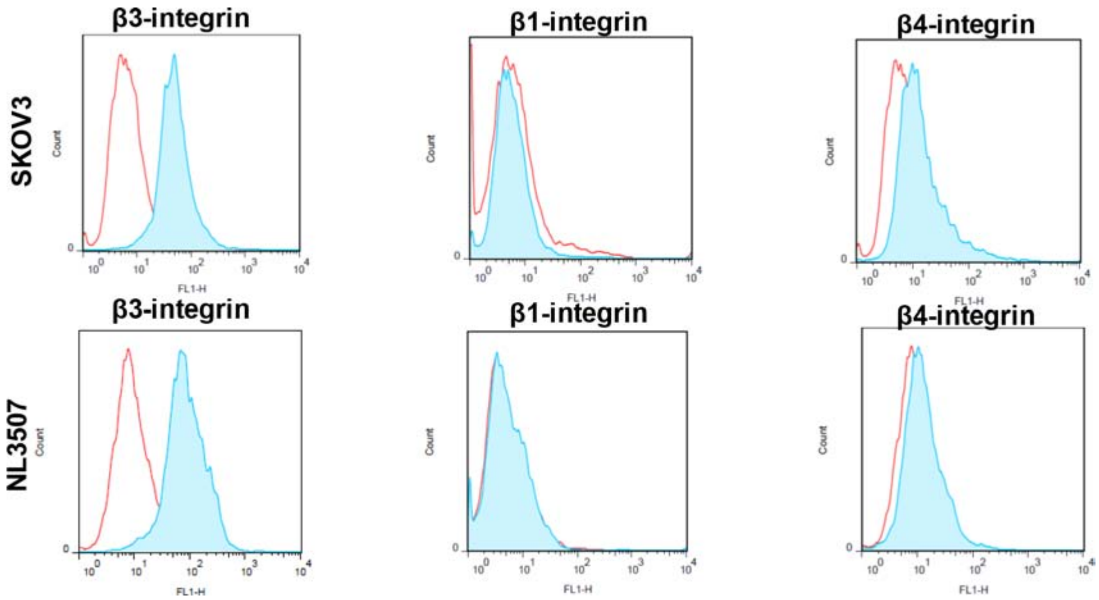

Supplementary Figure S3: Membrane expression of the integrin β3, β1 and β4 receptors was determined by flow cytometry on SKOV3 and NL3507 cells. The red and light blue peaks, respectively, represent the fluorescence of the cells incubated with the secondary antibody alone, as control (anti-mouse or-goat), and the anti-integrin Abs reported on the top.

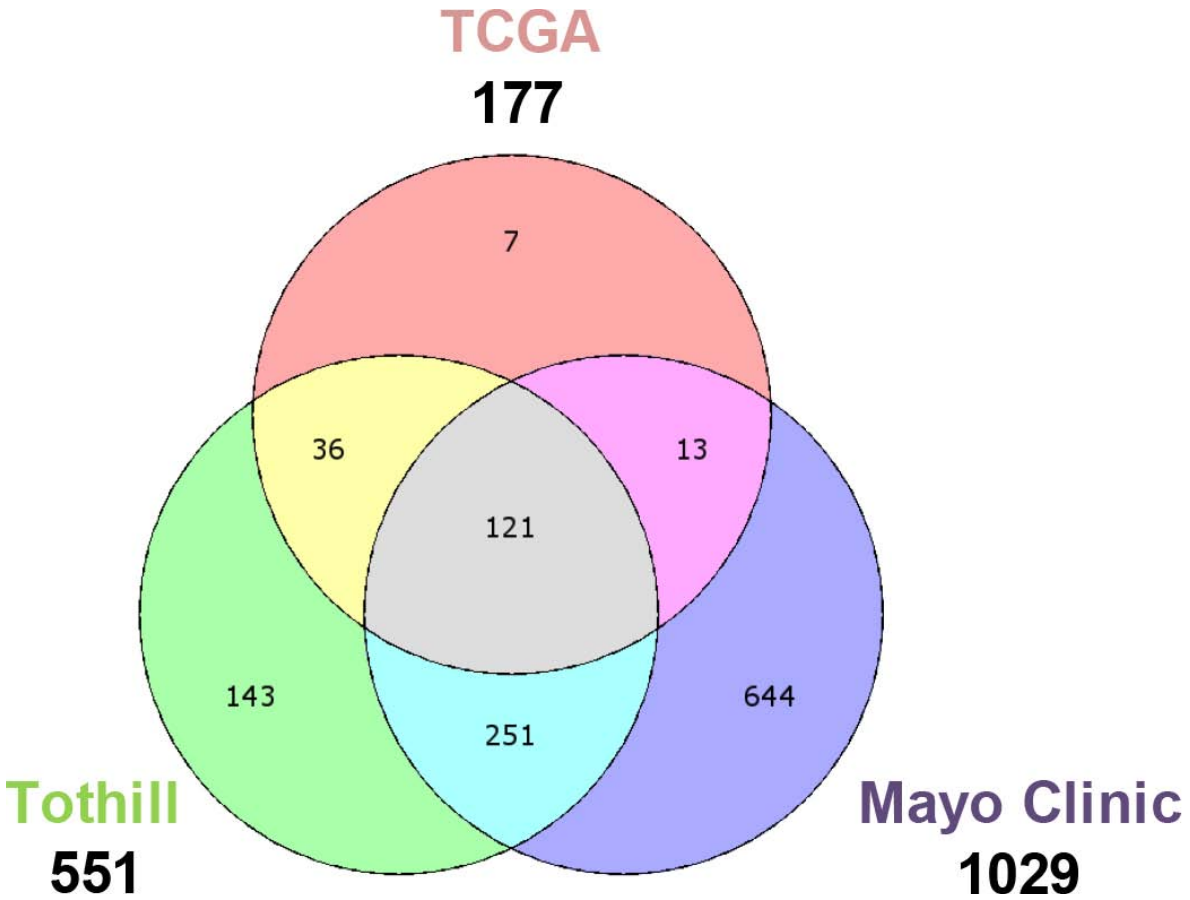

Supplementary Figure S4: Venn diagram of Axl-correlated genes obtained by analysis of three microarray datasets reported in the figure. The list of the 121 overlapping gene is reported in Table 1 and Supplementary Table 2.

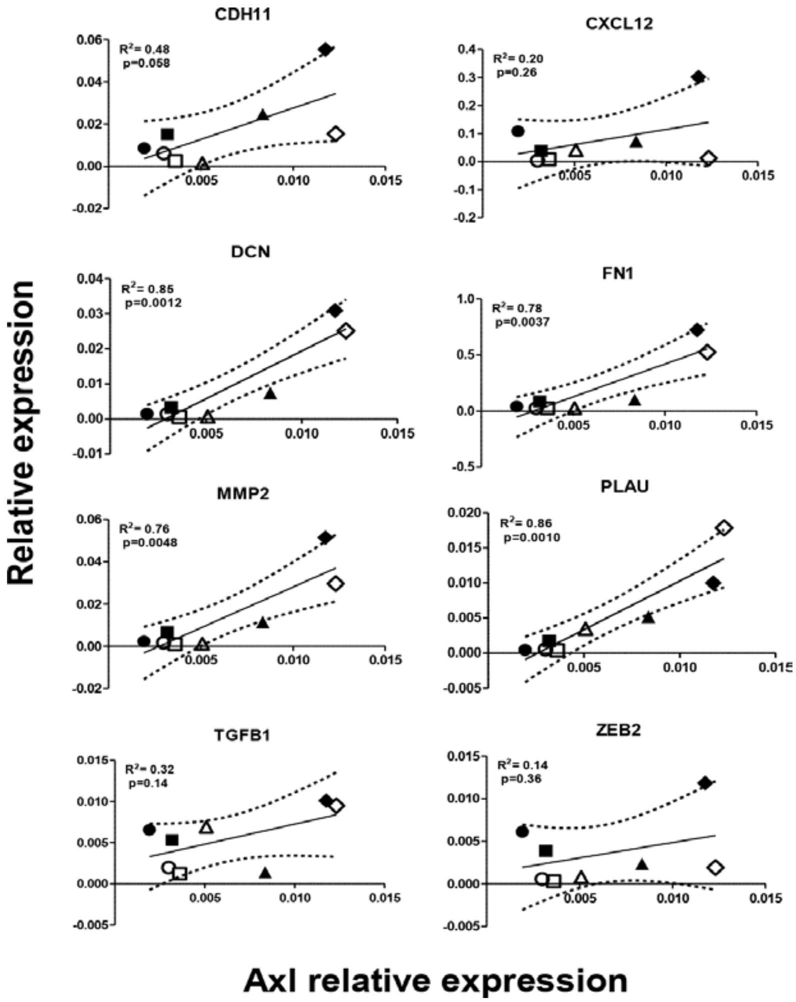

Supplementary Figure S5: Real time RT-PCR performed on total RNA extracted from eight HGEOC samples. The scatter plots show the correlation between Axl and co-regulated genes at the mRNA level. RNA was extracted from fresh frozen tissues (empty dots) or from FFPE samples (solid dots). Pearson's correlation of each probe and Axl was performed.

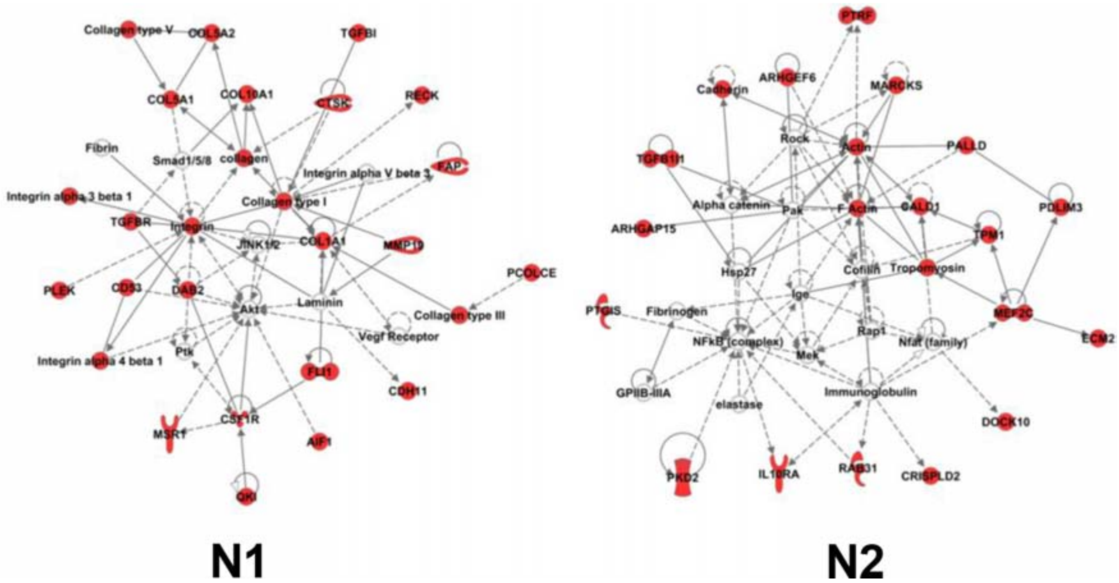

Supplementary Figure S6: Graphical representation of the top two networks (listed in Supplementary Table 5) identified by IPA. These networks were identified by loading the Axl-associated genes of Table 1. Axl-correlated genes included in the network are highlighted in red.

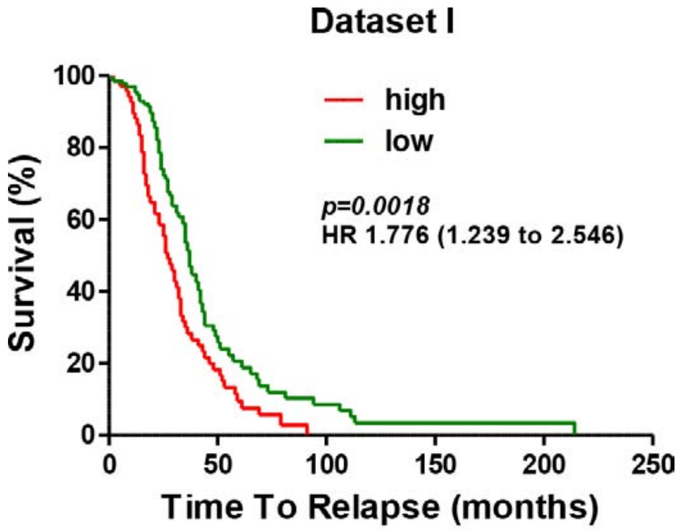

Metagene Expression	Median Survival (month)
high	27.00
low	37.00

Supplementary Figure S7: Kaplan-Meyer curves, long-rank *p* value and HR are to compare relapse free survival between HGEOC patients with 'high' (red lines) and 'low' (green lines expression intensities of the metagene consisting of Axl-driven signature. The tables below each curve reports the median survival of each group of patients.

Supplementary Table S1: IHC for Axl and p130cas performed on archival case material of 72 primary EOCs.

Clinicopathological characteristics	Total	(%)	AXL positive cases	(%)	AXL negative cases	%	p130 cas positive cases	(%)	p130 cas negative cases	(%)
**Tumor stage**										
I	19	26.39	12	63.16	7	36.84	13	68.42	6	31.58
II	6	8.33	5	83.33	1	16.67	4	66.67	2	33.33
III	47	65.28	41	87.23	6	12.77	42	89.36	5	10.64
**Tumor grade**										
1	10	13.89	5	50	5	50	5	50	5	50
2	23	31.94	16	69.57	7	30.43	20	86.96	3	13.04
3	37	51.39	35	94.59	2	5.41	32	86.49	5	13.51
Undifferentiated	2	2.78	2	100	0	0	2	100	0	0
**Histologic subtype**										
LGEC	4	5.56	3	75	1	25	3	75	1	25
MOC	10	13.89	2	20	8	80	3	30	7	70
COC	2	2.78	2	100	0	0	2	100	0	0
HGSC	51	70.83	46	90.2	5	9.8	46	90.2	5	9.8
HGEC	3	4.17	3	100	0	0	3	100	0	0
Undifferentiated	2	2.78	2	100	0	0	2	100	0	0
**Tumor Type**										
Type I	16	22.22	7	43.75	9	56.25	8	50	8	50
Type II	56	77.78	51	91.07	5	8.93	51	91.07	5	8.93

Supplementary Table S2: List of the data sets reporting the gene expression profiles analyzed in the present study.

Datasets	Platform	Array	No. of probes	No. of ovarian cancer patients
I (Tothill 2008)	Affymetrix	HG-U133 Plus 2	54675	198
II (TCGA 2011)	Affymetrix	HT_HG-U133A	22277	578
III (Mayo Clinic 2014)	Agilent	4x44K	27958	174

Supplementary Table S3: Axl-correlated genes common to the three analyzed datasets.

GeneSymbol	Gene tytle	Dataset I			Dataset II			Dataset III		
		ProbeID	Correlation	*p*.value	ProbeID	Correlation	*p*.value	ProbeID	Correlation	*p*.value
**ACTA2**	actin, alpha 2, smooth muscle, aorta	200974_at	0.464314787	2.65E-12	200974_at	0.448358728	0	A_23_P150053	0.633623383	0
**ADAM12**	ADAM metallopeptidase domain 12	226777_at	0.495216605	5.08E-14	202952_s_at	0.422823374	0	A_23_P202327	0.566203878	4.44E-16
**AEBP1**	AE binding protein 1	201792_at	0.559310854	0	201792_at	0.452633831	0	A_23_P145916	0.641612394	0
AIF1	allograft inflammatory factor 1	213095_x_at	0.488581332	1.23E-13	209901_x_at	0.450162071	0	A_23_P214627	0.418103024	9.44E-09
ALOX5AP	arachidonate 5-lipoxygenase-activating protein	204174_at	0.400850876	2.83E-09	204174_at	0.45239476	0	A_24_P347378	0.576569242	0
**ANGPTL2**	angiopoietin-like 2	213001_at	0.50262846	1.87E-14	213001_at	0.400780866	0	A_23_P20864	0.491822188	5.51E-12
ARHGAP15	Rho GTPase activating protein 15	218870_at	0.45312164	1.01E-11	218870_at	0.404682428	0	A_23_P84154	0.465836577	9.33E-11
ARHGEF6	Rac/Cdc42 guanine nucleotide exchange factor (GEF) 6	209539_at	0.457323208	6.15E-12	209539_at	0.539581628	0	A_24_P228875	0.588544657	0
ATP6V1B2	ATPase, H+ transporting, lysosomal 56/58kDa, V1 subunit B2	201089_at	0.426947525	1.91E-10	201089_at	0.408547394	0	A_23_P31844	0.474015113	3.93E-11
ATP8B4	ATPase, class I, type 8B, member 4	220416_at	0.435208813	7.77E-11	220416_at	0.404609231	0	A_23_P163216	0.558795591	1.11E-15
**AXL**	AXL receptor tyrosine kinase	202686_s_at	1	0	202686_s_at	1	0	A_23_P208389	1	0
C1QA	complement component 1, q subcomponent, A chain	218232_at	0.426498686	2.01E-10	218232_at	0.424296311	0	A_24_P222655	0.460542515	1.61E-10

(*Continued*)

GeneSymbol	Gene tytle	Dataset I			Dataset II			Dataset III		
		ProbeID	Correlation	*p*.value	ProbeID	Correlation	*p*.value	ProbeID	Correlation	*p*.value
C1QB	complement component 1, q subcomponent, B chain	202953_at	0.459449202	4.77E-12	202953_at	0.445158722	0	A_23_P137366	0.483820106	1.35E-11
CALD1	caldesmon 1	212077_at	0.516428818	2.66E-15	212077_at	0.414564156	0	A_24_P921366	0.753611066	0
**CD14**	CD14 molecule	201743_at	0.474773866	7.27E-13	201743_at	0.443959153	0	A_24_P283189	0.535123003	2.80E-14
CD53	CD53 molecule	203416_at	0.457393313	6.10E-12	203416_at	0.430803285	0	A_23_P74547	0.562339958	6.66E-16
CD84	CD84 molecule	230391_at	0.481378253	3.14E-13	205988_at	0.422139419	0	A_23_P361940	0.461299325	1.49E-10
CD93	CD93 molecule	202878_s_at	0.467040783	1.90E-12	202878_s_at	0.510777107	0	A_32_P56001	0.620685459	0
**CDH11**	cadherin 11, type 2, OB-cadherin	239286_at	0.482927278	2.57E-13	207172_s_at	0.444722548	0	A_23_P152305	0.657586716	0
**COL10A1**	collagen, type X, alpha 1	217428_s_at	0.512489328	4.66E-15	217428_s_at	0.419132254	0	A_23_P214144	0.5024193	1.62E-12
**COL10A2**	collagen, type I, alpha 1	202311_s_at	0.523970103	8.88E-16	217430_x_at	0.454204481	0	A_23_P207520	0.574026737	2.22E-16
**COL10A3**	collagen, type V, alpha 1	203325_s_at	0.54450622	0	212489_at	0.425198078	0	A_23_P158593	0.603060312	0
**COL10A4**	collagen, type V, alpha 2	221729_at	0.550757587	0	221730_at	0.40010105	0	A_23_P33196	0.655245341	0
**COL10A5**	collectin sub-family member 12	221019_s_at	0.520255424	1.55E-15	221019_s_at	0.419164615	0	A_23_P27306	0.645145657	0
**COL10A6**	coatomer protein complex, subunit zeta 2	219561_at	0.518928515	1.78E-15	219561_at	0.428773139	0	A_23_P101093	0.54036479	1.40E-14
**COL10A7**	cysteine-rich secretory protein LCCL domain containing 2	221541_at	0.548385826	0	221541_at	0.447917016	0	A_23_P106602	0.588790178	0

(*Continued*)

GeneSymbol	Gene tytle	Dataset I			Dataset II			Dataset III		
		ProbeID	Correlation	*p*.value	ProbeID	Correlation	*p*.value	ProbeID	Correlation	*p*.value
**CSF1R**	colony stimulating factor 1 receptor	203104_at	0.507043717	9.99E-15	203104_at	0.488186272	0	A_23_P110791	0.608084568	0
CSGALNACT2	chondroitin sulfate N-acetylgalactosaminyl transferase 2	222235_s_at	0.467482858	1.80E-12	222235_s_at	0.415166658	0	A_23_P149892	0.678845758	0
CTSK	cathepsin K	202450_s_at	0.582258228	0	202450_s_at	0.445233509	0	A_23_P34744	0.592429376	0
**CXCL12**	chemokine (C-X-C motif) ligand 12	203666_at	0.501256344	2.24E-14	209687_at	0.451314293	0	A_24_P412156	0.634848934	0
**CYBB**	cytochrome b-245, beta polypeptide	203923_s_at	0.4632151	3.03E-12	203923_s_at	0.490566916	0	A_24_P365767	0.568344033	2.22E-16
DAB2	disabled homolog 2, mitogen-responsive phosphoprotein (Drosophila)	210757_x_at	0.622552414	0	201279_s_at	0.605212278	0	A_23_P257871	0.69517025	0
**DCN**	decorin	209335_at	0.495955431	4.60E-14	211896_s_at	0.459153214	0	A_23_P64873	0.600657916	0
**DOCK10**	dedicator of cytokinesis 10	219279_at	0.473283732	8.76E-13	219279_at	0.485959679	0	A_23_P16722	0.527827499	7.22E-14
DOCK2	dedicator of cytokinesis 2	213160_at	0.48877907	1.20E-13	213160_at	0.487188811	0	A_24_P408704	0.476624952	2.97E-11
ECM2	extracellular matrix protein 2, female organ and adipocyte specific	206101_at	0.468992386	1.49E-12	206101_at	0.442002578	0	A_23_P303671	0.583934161	0
**EDNRA**	endothelin receptor type A	204464_s_at	0.557367824	0	216235_s_at	0.400134427	0	A_24_P217572	0.590533274	0
**EMP3**	epithelial membrane protein 3	203729_at	0.539658353	0	203729_at	0.532199137	0	A_23_P119362	0.430678476	3.00E-09
**ENTPD1**	ectonucleoside triphosphate diphosphohydrolase 1	209473_at	0.515450649	3.11E-15	209473_at	0.487862895	0	A_23_P24260	0.572058392	2.22E-16

(*Continued*)

GeneSymbol	Gene tytle	Dataset I			Dataset II			Dataset III		
		ProbeID	Correlation	*p*.value	ProbeID	Correlation	*p*.value	ProbeID	Correlation	*p*.value
EPB41L3	erythrocyte membrane protein band 4.1-like 3	211776_s_at	0.489108432	1.15E-13	206710_s_at	0.502619332	0	A_23_P4536	0.573460791	2.22E-16
EVI2A	ecotropic viral integration site 2A	204774_at	0.552338452	0	204774_at	0.467549403	0	A_23_P78092	0.510037816	6.55E-13
**EVI2B**	ecotropic viral integration site 2B	211742_s_at	0.467085075	1.89E-12	211742_s_at	0.478524253	0	A_23_P66694	0.417564867	9.90E-09
FAP	fibroblast activation protein, alpha	209955_s_at	0.553238869	0	209955_s_at	0.426747243	0	A_23_P56746	0.586666676	0
**FBN1**	fibrillin 1	202766_s_at	0.567052708	0	202766_s_at	0.493085809	0	A_23_P65678	0.612614725	0
**FCGR2B**	Fc fragment of IgG, low affinity IIb, receptor (CD32)	210889_s_at	0.435161656	7.81E-11	210889_s_at	0.403864207	0	A_23_P34644	0.535136089	2.80E-14
FCGR3B	Fc fragment of IgG, low affinity IIIb, receptor (CD16b)	204007_at	0.414423295	7.18E-10	204007_at	0.411599745	0	A_23_P126298	0.57473827	0
FLI1	Friend leukemia virus integration 1	204236_at	0.520668853	1.33E-15	204236_at	0.519697751	0	A_24_P355649	0.530591585	5.06E-14
**FN1**	fibronectin 1	210495_x_at	0.521968868	1.11E-15	212464_s_at	0.468950478	0	A_24_P119745	0.588786559	0
**FSTL1**	follistatin-like 1	208782_at	0.449742759	1.50E-11	208782_at	0.443452165	0	A_23_P212696	0.638741327	0
FZD1	frizzled homolog 1 (Drosophila)	204451_at	0.534536484	2.22E-16	204451_at	0.416546549	0	A_23_P134502	0.597849677	0
GIMAP6	GTPase, IMAP family member 6	219777_at	0.494905446	5.31E-14	219777_at	0.468021191	0	A_23_P145631	0.499419386	2.30E-12
GLIPR1	GLI pathogenesis-related 1	204222_s_at	0.540558851	0	204222_s_at	0.613415396	0	A_23_P364024	0.491607291	5.65E-12
GLT8D2	glycosyltransferase 8 domain containing 2	227070_at	0.52501987	6.66E-16	221447_s_at	0.482996653	0	A_23_P48198	0.662586761	0

(*Continued*)

GeneSymbol	Gene tytle	Dataset I			Dataset II			Dataset III		
		ProbeID	Correlation	*p*.value	ProbeID	Correlation	*p*.value	ProbeID	Correlation	*p*.value
**GPR65**	G protein-coupled receptor 65	214467_at	0.476166315	6.10E-13	214467_at	0.423633909	0	A_32_P65804	0.546221045	6.44E-15
HCK	hemopoietic cell kinase	208018_s_at	0.456556947	6.73E-12	208018_s_at	0.418150859	0	A_24_P365526	0.448024808	5.68E-10
HEG1	HEG homolog 1 (zebrafish)	213069_at	0.553825248	0	213069_at	0.488976885	0	A_32_P166693	0.620229555	0
**HEPH**	hephaestin	203903_s_at	0.465868429	2.19E-12	203903_s_at	0.429170323	0	A_24_P399980	0.550403094	3.55E-15
IFFO1	intermediate filament family orphan 1	36030_at	0.57926879	0	36030_at	0.532416728	0	A_23_P87742	0.662322146	0
**IL10RA**	interleukin 10 receptor, alpha	204912_at	0.463855635	2.80E-12	204912_at	0.490902458	0	A_23_P203173	0.571108996	2.22E-16
ITGA4	integrin, alpha 4 (antigen CD49)	213416_at	0.516386742	2.66E-15	213416_at	0.479064627	0	A_23_P56505	0.481980019	1.66E-11
LAIR1	leukocyte-associated immunoglobulin-like receptor 1	210644_s_at	0.452307282	1.11E-11	210644_s_at	0.435871344	0	A_23_P209135	0.534532342	3.04E-14
LAPTM5	lysosomal protein transmembrane 5	201721_s_at	0.461211535	3.86E-12	201720_s_at	0.44167746	0	A_23_P86283	0.562621585	6.66E-16
LAT2	linker for activation of T cells family, member 2	221581_s_at	0.492215203	7.59E-14	221581_s_at	0.408868671	0	A_24_P351852	0.41003077	1.92E-08
LCP2	lymphocyte cytosolic protein 2	205269_at	0.453244836	9.95E-12	205270_s_at	0.464491675	0	A_23_P30547	0.444467838	8.05E-10
LHFP	lipoma HMGIC fusion partner	218656_s_at	0.520984117	1.33E-15	218656_s_at	0.458981976	0	A_23_P88069	0.601968974	0
**LHFPL2**	lipoma HMGIC fusion partner-like 2	212658_at	0.418815204	4.54E-10	212658_at	0.491391525	0	A_23_P255104	0.535216027	2.78E-14

(*Continued*)

GeneSymbol	Gene tytle	Dataset I			Dataset II			Dataset III		
		ProbeID	Correlation	*p*.value	ProbeID	Correlation	*p*.value	ProbeID	Correlation	*p*.value
MAF	v-maf musculoaponeurotic fibrosarcoma oncogene homolog (avian)	209348_s_at	0.520469063	1.55E-15	209348_s_at	0.452857031	0	A_24_P256219	0.571717169	2.22E-16
MAFB	v-maf musculoaponeurotic fibrosarcoma oncogene homolog B (avian)	218559_s_at	0.465703448	2.24E-12	218559_s_at	0.429883246	0	A_23_P17345	0.535586473	2.64E-14
MARCH1	membrane-associated ring finger (C3HC4) 1	219574_at	0.521102877	1.33E-15	219574_at	0.420845974	0	A_24_P354412	0.573012843	2.22E-16
**MARCKS**	myristoylated alanine-rich protein kinase C substrate	225897_at	0.5359927	2.22E-16	201669_s_at	0.47279083	0	A_23_P214222	0.66787418	0
**MEF2C**	myocyte enhancer factor 2C	209199_s_at	0.46105928	3.93E-12	209200_at	0.493000136	0	A_23_P320739	0.480522597	1.94E-11
MFSD1	major facilitator superfamily domain containing 1	218109_s_at	0.403911124	2.09E-09	218109_s_at	0.414674714	0	A_23_P166677	0.588066874	0
MMP19	matrix metallopeptidase 19	204575_s_at	0.478294504	4.66E-13	204575_s_at	0.410183901	0	A_23_P203882	0.671919851	0
**MMP2**	matrix metallopeptidase 2	201069_at	0.555883345	0	201069_at	0.489714586	0	A_23_P163787	0.515381302	3.42E-13
**MNDA**	myeloid cell nuclear differentiation antigen	204959_at	0.508180748	8.66E-15	204959_at	0.489237022	0	A_23_P137935	0.545783492	6.88E-15
MS4A4A	membrane-spanning 4-domains	1555728_a_at	0.435330294	7.67E-11	219607_s_at	0.475077184	0	A_23_P75769	0.562552056	6.66E-16
MS4A6A	membrane-spanning 4-domains	230550_at	0.506861054	1.02E-14	219666_at	0.487733896	0	A_23_P36120	0.561322409	8.88E-16
MSR1	macrophage scavenger receptor 1	214770_at	0.543395958	0	214770_at	0.437967608	0	A_24_P372223	0.519978946	1.94E-13
MXRA8	matrix-remodelling associated 8	213422_s_at	0.436372942	6.83E-11	213422_s_at	0.406469674	0	A_23_P32444	0.541026104	1.29E-14

(Continued)

GeneSymbol	Gene tytle	Dataset I			Dataset II			Dataset III		
		ProbeID	Correlation	*p*.value	ProbeID	Correlation	*p*.value	ProbeID	Correlation	*p*.value
**NPL**	N-acetylneuraminate pyruvate lyase	221210_s_at	0.426806984	1.94E-10	221210_s_at	0.413354648	0	A_23_P381431	0.444950653	7.68E-10
OLFML2B	olfactomedin-like 2B	213125_at	0.524303096	8.88E-16	213125_at	0.440584923	0	A_24_P248345	0.530028498	5.44E-14
**OLFML3**	olfactomedin-like 3	218162_at	0.614535893	0	218162_at	0.599938096	0	A_24_P11315	0.625704942	0
**PALLD**	palladin, cytoskeletal associated protein	200897_s_at	0.446178766	2.26E-11	200897_s_at	0.471784236	0	A_23_P213102	0.526627065	8.39E-14
**PCOLCE**	procollagen C-endopeptidase enhancer	202465_at	0.488488022	1.25E-13	202465_at	0.437724947	0	A_23_P251499	0.570668613	2.22E-16
**PDLIM3**	PDZ and LIM domain 3	209621_s_at	0.523133639	8.88E-16	209621_s_at	0.452218806	0	A_23_P110403	0.586865736	0
**PDPN**	podoplanin	204879_at	0.472161368	1.01E-12	204879_at	0.42922032	0	A_24_P299685	0.505084512	1.18E-12
PECAM1	platelet/endothelial cell adhesion molecule	208981_at	0.451516018	1.22E-11	208983_s_at	0.503926378	0	A_23_P252471	0.41468293	1.28E-08
PKD2	polycystic kidney disease 2	203688_at	0.436429398	6.79E-11	203688_at	0.433863048	0	A_24_P106112	0.685967606	0
**PLAU**	plasminogen activator, urokinase	205479_s_at	0.529198935	4.44E-16	211668_s_at	0.411913147	0	A_23_P24104	0.603868878	0
**PLEK**	pleckstrin	203471_s_at	0.413908764	7.57E-10	203471_s_at	0.422255276	0	A_23_P209678	0.472739201	4.50E-11
PLXDC1	plexin domain containing 1	219700_at	0.511472677	5.33E-15	219700_at	0.400870064	0	A_32_P208823	0.49325781	4.68E-12
**PLXNC1**	plexin C1	213241_at	0.5192909	1.78E-15	213241_at	0.524331652	0	A_24_P196351	0.583607799	0
**PMP22**	peripheral myelin protein 22	210139_s_at	0.534084127	2.22E-16	210139_s_at	0.475572559	0	A_23_P100711	0.728995605	0
**PTGER4**	prostaglandin E receptor 4	204897_at	0.452076588	1.14E-11	204897_at	0.437790422	0	A_23_P148047	0.616650796	0

(*Continued*)

GeneSymbol	Gene tytle	Dataset I			Dataset II			Dataset III		
		ProbeID	Correlation	*p*.value	ProbeID	Correlation	*p*.value	ProbeID	Correlation	*p*.value
PTGIS	prostaglandin I2 (prostacyclin) synthase	208131_s_at	0.504377323	1.47E-14	208131_s_at	0.420057457	0	A_24_P48723	0.621722406	0
**PTPRC**	protein tyrosine phosphatase, receptor type, C	212588_at	0.476185671	6.08E-13	207238_s_at	0.462511604	0	A_23_P125451	0.478341039	2.46E-11
PTRF	polymerase I and transcript release factor	208790_s_at	0.52577354	6.66E-16	208790_s_at	0.450485689	0	A_23_P394064	0.620366578	0
**QKI**	quaking homolog, KH domain RNA binding (mouse)	212636_at	0.610260079	0	212636_at	0.51016637	0	A_24_P940426	0.646206959	0
RAB31	RAB31, member RAS oncogene family	217763_s_at	0.594583853	0	217764_s_at	0.527178147	0	A_24_P236799	0.736208955	0
RECK	reversion-inducing-cysteine-rich protein with kazal motifs	205407_at	0.453143433	1.01E-11	205407_at	0.431304922	0	A_23_P83028	0.646647682	0
**RGS4**	regulator of G-protein signaling 4	204337_at	0.433291699	9.60E-11	204337_at	0.407112142	0	A_23_P200737	0.52294978	1.34E-13
**RNASE6**	ribonuclease, RNase A family, k6	213566_at	0.466205644	2.10E-12	213566_at	0.498987392	0	A_23_P3014	0.460634622	1.60E-10
SAMSN1	SAM domain, SH3 domain and nuclear localization signals 1	220330_s_at	0.451467917	1.22E-11	220330_s_at	0.404285033	0	A_23_P29005	0.552482237	2.66E-15
SERPINF1	serpin peptidase inhibitor, clade F	202283_at	0.586869296	0	202283_at	0.482439594	0	A_23_P100660	0.652981095	0
**SFRP4**	secreted frizzled-related protein 4	204051_s_at	0.429081295	1.52E-10	204051_s_at	0.460908612	0	A_23_P215328	0.586678248	0
**SH2B3**	SH2B adaptor protein 3	203320_at	0.509127208	7.55E-15	203320_at	0.524650147	0	A_24_P201739	0.619266178	0
SIRPA	signal-regulatory protein alpha	202897_at	0.525695503	6.66E-16	202897_at	0.427392697	0	A_24_P259083	0.620057475	0
SNAI2	snail homolog 2 (Drosophila)	213139_at	0.549013935	0	213139_at	0.478505265	0	A_24_P937139	0.442942025	9.34E-10

(*Continued*)

GeneSymbol	Gene tytle	Dataset I			Dataset II			Dataset III		
		ProbeID	Correlation	*p*.value	ProbeID	Correlation	*p*.value	ProbeID	Correlation	*p*.value
SPATS2L	spermatogenesis associated, serine-rich 2-like	215617_at	0.58915976	0	215617_at	0.44295441	0	A_23_P131255	0.41702206	1.04E-08
**TGFB1**	transforming growth factor, beta 1	203085_s_at	0.556386038	0	203085_s_at	0.469708365	0	A_24_P79054	0.401922248	3.86E-08
TGFB1I1	transforming growth factor beta 1 induced transcript 1	209651_at	0.517789621	2.22E-15	209651_at	0.428802113	0	A_23_P141055	0.530118454	5.37E-14
**TGFBI**	transforming growth factor, beta-induced, 68kDa	201506_at	0.515232229	3.11E-15	201506_at	0.513947319	0	A_23_P156327	0.62789459	0
**TGFBR2**	transforming growth factor, beta receptor II (70/80kDa)	208944_at	0.446282695	2.23E-11	208944_at	0.489943954	0	A_23_P211957	0.697262955	0
**TIMP3**	TIMP metallopeptidase inhibitor 3	201150_s_at	0.52546818	6.66E-16	201149_s_at	0.485036	0	A_23_P399078	0.59241473	0
**TLR7**	toll-like receptor 7	220146_at	0.546012188	0	220146_at	0.501537874	0	A_23_P85240	0.601047609	0
TM6SF1	transmembrane 6 superfamily member 1	219892_at	0.475689681	6.48E-13	219892_at	0.481566786	0	A_23_P77117	0.659053481	0
TPM1	tropomyosin 1 (alpha)	210986_s_at	0.464672816	2.54E-12	210987_x_at	0.402152415	0	A_23_P206018	0.697654327	0
VSIG4	V-set and immunoglobulin domain containing 4	204787_at	0.472295243	9.92E-13	204787_at	0.430632369	0	A_23_P217269	0.535123392	2.80E-14
ZCCHC24	zinc finger, CCHC domain containing 24	212423_at	0.582976718	0	212419_at	0.502919618	0	A_23_P335452	0.445469367	7.30E-10
ZEB1	zinc finger E-box binding homeobox 1	212764_at	0.574168423	0	212764_at	0.539658895	0	A_23_P202013	0.47202727	4.86E-11
**ZEB2**	zinc finger E-box binding homeobox 2	203603_s_at	0.54924819	0	203603_s_at	0.441971407	0	A_23_P142560	0.662647295	0

Supplementary Table S4: Characteristics of the EOC patients evaluated in the present study.

Sample ID	Histotype	Grading	Ascites: presence of:		
			Tumor cells[a]	Immune cells[a]	Mesothelial cells[a]
1	Serous	G3	Abundant	Present	Present
2	Serous	G3	Abundant	Present	Present
3	Serous	G3	Present	Present	Present
4	Serous	G3	Abudant	Absent	Present
5	Serous	G3	Abudant	Absent	Present
6	Serous	G3	Present	–	Present
7	Serous	G3	Abundant	Present	–
8	Serous	G3	Abundant	–	–
9	Serous	G3	Abundant	–	Present
10	Serous	G3	Abundant	Present	Present

[a]The amount of cells present in ascites of EOC patients as defined by the cytopathologist at diagnosis.

Supplementary Table S5A: IPA results obtained with the Axl-correlated gene set.

Molecular and Cellular Functions

Name	*p*-value	Molecules
Cellular Movement	*6.22E-05 – 1.27E-20*	61
Cell-To-Cell Signaling and Interaction	*4.83E-05 – 7.85E-18*	57
Cellular Development	*4.72E-05 – 1.03E-16*	65
Cellular Growth and Proliferation	*6.12E-05 – 1.03E-16*	71
Cell Morphology	*3.52E-05 – 3.34E-16*	61

Network

#	Name	Score	Focus Molecules
1	Connective Tissue Disorders, Dermatological Diseases and Conditions, Developmental Disorder	33	18
2	Cancer, Organismal Injury and Abnormalities, Reproductive System Disease Cell-To-Cell Signaling and Interaction, Hematological System Development and Function, Immune Cell,Trafficking	33	19
3	Cell-To-Cell Signaling and Interaction, Hematological System Development and Function, Immune Cell,Trafficking	32	17
4	Cell Cycle, Cell Death and Survival, Hematological System Development and Function	26	16
5	Cell-To-Cell Signaling and Interaction, Tissue Development, Hematological System Development and Function	24	14

Supplementary Table S5B: IPA results obtained with the Axl-driven signature.

Network

#	Name	Score	Focus Molecules
1	Tissue Morphology, Cell Morphology, Cellular Assembly and Organization	31	15
2	Cancer, Cellular Development, Organismal Injury and Abnormalities	22	12
3	Cellular Function and Maintenance, Hematological System Development and Function, Cell-To-Cell Signaling and Interaction	21	11
4	Cardiovascular Disease, Cell-To-Cell Signaling and Interaction, Hematological System Development and Function	17	9
5	Cancer, Organismal Injury and Abnormalities, Reproductive System Disease	13	8

Supplementary Table S6: List of the probes used for the real time RT-PCR.

Gene	ID Assay
AXL	Hs01064444_m1
MERTK	Hs00179024_m1
TYRO3	Hs00170723_m1
GAS6	Hs01090305_m1
CDH11	Hs00901475_m1
CXCL12	Hs00171022_m1
DCN	Hs00370384_m1
FN1	Hs00277509_m1
GAPDH	Hs03929097_g1
MMP2	Hs01548727_m1
PLAU	Hs00170182_m1
TGFB1	Hs99999918_m1
ZEB2	Hs00207691_m1

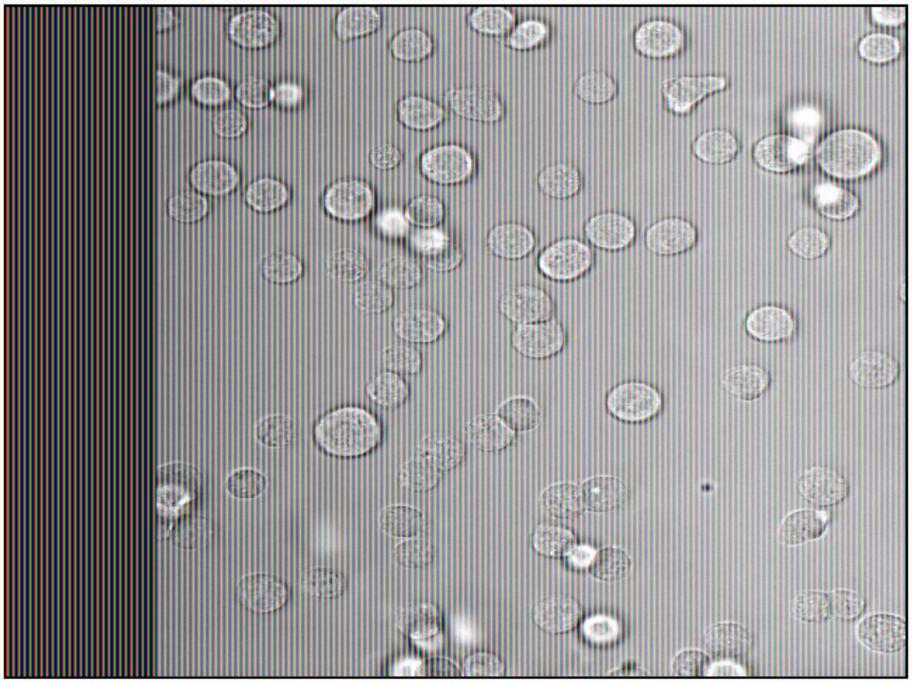

Supplementary Figure S1: Video reporting the live cell imaging performed on Gas6-stimulated control siRNA transfected SKOV3 cells during adhesion on FN.

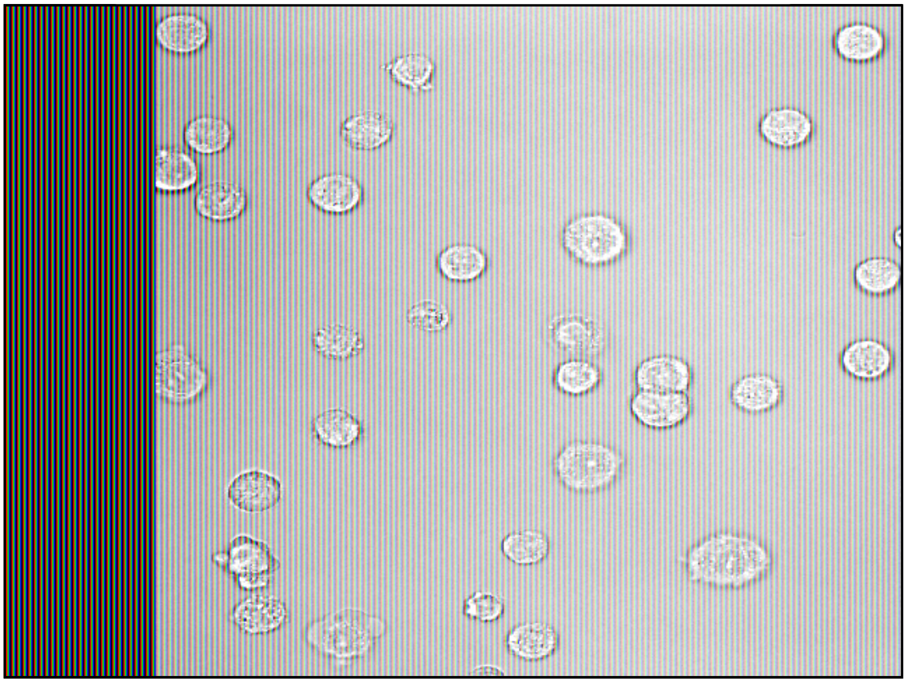

Supplementary Figure S2: Video reporting the live cell imaging performed on Gas6-stimulated p130cas siRNA transfected SKOV3 cells during adhesion on FN.
